# Multi-omics reveals efferocytosis-related hub genes as biomarkers for ustekinumab response in colitis

**DOI:** 10.3389/fimmu.2025.1597528

**Published:** 2025-09-19

**Authors:** Jun-meng Wang, Wan-yu Xia, Yu-sha Liao, Jing Yuan

**Affiliations:** Acupuncture and Tuina School, Chengdu University of Traditional Chinese Medicine, Chengdu, Sichuan, China

**Keywords:** efferocytosis, ulcerative colitis, multi-omics integration, ustekinumab response, myeloid-stromal crosstalk

## Abstract

**Background:**

Defective efferocytosis in ulcerative colitis (UC) exacerbates inflammation due to impaired clearance of apoptotic cells, yet the molecular mechanisms linking efferocytosis-related genes to therapeutic outcomes remain unclear. This study aims to investigate the role of efferocytosis in UC and the key regulatory mechanism of efferocytosis.

**Methods:**

Multi-omics integration of single-cell and bulk transcriptomic data from human UC colonic mucosa identified efferocytosis-active cellular subpopulations. Machine learning algorithms screened hub genes, followed by molecular docking to assess interactions with UST. A mouse colitis model was used to verify the inflammatory damage of UC and the key genes that play the role in efferocytosis.

**Results:**

In UC, the “eat me” and “digest me” signaling pathways are predominantly upregulated in myeloid cells, while the “find me” signaling cascade shows marked activation in stromal cells. Macrophages characterized by the M2 polarization demonstrate enhanced phagocytic proficiency and are instrumental in the engulfment and clearance of apoptotic cells, thereby alleviating the inflammatory cascade in UC. Six hub genes (ANXA1, PANX1, ANXA5, CD93, SERPINE1, MFGE8) were associated with UC progression and correlated with clinical response to UST. Molecular docking analysis revealed strong binding affinities between these gene-encoded proteins and UST. Transcriptomic and proteomic analyses confirmed dysregulated expression of these hub genes in the colitis model.

**Conclusion:**

This study reveals cellular heterogeneity at different stages of efferocytosis in UC, identifies efferocytosis-related genes as critical regulators of mucosal repair and predictors of UST efficacy. Findings emphasize targeting macrophage-driven efferocytosis to resolve inflammation, offering novel strategies for improving treatment outcomes.

## 1 Introduction

Diffuse and recurrent mucosal inflammation is the key pathogenesis of ulcerative colitis (UC). Recurrent episodes of colonic inflammation lead to intestinal mucosal damage, manifesting as diarrhea, hematochezia, and other pathological alterations in patients (1). The suppression of the inflammatory response is pivotal for the restoration of the intestinal mucosal integrity and has emerged as a critical target for the development of innovative anti-colitis therapeutics (2, 3).

Efferocytosis is a pivotal intrinsical anti-inflammatory mechanism that suppresses excessive inflammation by rapidly clearing apoptotic cells via sequential “find me,” “eat me,” “don’t eat me,” and “digest me” signals. Efferocytosis prevents the release of damage-associated molecular patterns (DAMPs) by efficiently clearing dead or injured cells, thereby repressing exacerbated inflammatory cascades (4, 5). Increasing efferocytosis has been shown as a promising approach to alleviate inflammation (6). Defective efferocytosis exacerbates mucosal damage in UC by accumulating apoptotic epithelial cells, which perpetuate inflammation and impair barrier repair (7, 8). Therefore, elucidating the functional significance of efferocytosis in UC pathogenesis and deciphering its molecular regulatory network are essential for developing novel therapeutic strategies targeting inflammation resolution and mucosal repair in UC.

We used single-cell transcriptomic datasets from publicly available repositories to map the involvement of colonic cellular subpopulations in distinct phases of efferocytosis (“find-me,” “eat me,” “don,t eat me,” and “digest me”) in colitis patients. Our analysis revealed that M2-polarized macrophages exhibited the highest efferocytosis capacity. Furthermore, key hub genes were identified through bulk-RNA sequencing, and their correlation with patient responses to pharmacological treatments was elucidated by analyzing clinical drug response outcomes. By constructing a colitis animal model, we employed transcriptomic and proteomic sequencing to further validate the expression of the pivotal hub genes in the animal model. Our findings contribute to a more profound understanding of the underlying pathogenic mechanisms of UC and facilitate the identification of potential therapeutic targets for its treatment.

## 2 Methods

### 2.1 Datasets and human sample selection

We searched GEO databases using the keyword “ulcerative colitis,” the filter criteria were as follows: ① human; ② the dataset had at least five healthy control and five UC samples. Finally, one single-cell RNA sequencing dataset (GSE214695), eight mRNA datasets (GSE193677, GSE66407, GSE75214, GSE107499, GSE206285, GSE92415, GSE23597, GSE73661) were included (**Table 1**).

**Table 1:** The information of all the datasets in the study.

GEO number	Type	Tissue	Platform
GSE214695	scRNA	Colonic mucosal	GPL18573
GSE193677	mRNA-seq	Colonic mucosal	GPL16791
GSE66407	Array	Colonic mucosal	GPL19833
GSE75214	Array	Colonic mucosal	GPL16244
GSE107499	Array	Colonic mucosal	GPL15207
GSE87466	Array	Colonic mucosal	GPL13158
GSE206285	Array	Colonic mucosal	GPL13158
GSE92415	Array	Colonic mucosal	GPL13158
GSE23597	Array	Colonic mucosal	GPL570
GSE73661	Array	Colonic mucosal	GPL16244

### 2.2 Identification of efferocytosis-related gene list

The efferocytosis-related gene list was created by combining the gene lists of “find me”, “eat me”, “don’t eat me” and “digest me” of several references (**Supplementary Table 2**) (9–11).

### 2.3 Single cell RNA sequencing data analysis

Colonic mucosal scRNA-seq data from healthy controls (n=6) and UC patients (n=6) were integrated and analyzed. After quality control and doublet removal, we performed cell clustering and annotated major cell types (epithelial, immune, stromal). Efferocytosis activity was quantified for each cell type. Macrophage subtypes were identified, and their efferocytosis capacity was compared using pseudotime trajectory analysis (12). Detailed parameters in **Supplementary Data Sheet 1**.

### 2.4 Identification of differentially expressed genes of microarray and bulk RNA-seq

GEO2R (https://www.ncbi.nlm.nih.gov/geo/geo2r/) was used to obtain the genes expressed differently between UC samples and healthy samples from microarray datasets. The Benjamini and Hoch-berg false discovery rate and t-test methods were applied in the GEO2R tool to calculate the false discovery rate (FDR) and pvalue, respectively. Adjusted P-value < 0.05 and a | log_2_ (fold change) | ≥ log_2_ (1.5) were considered to be statistically significant. For datasets of RNA-seq, the raw counts files were downloaded and differential expression analysis was performed by DESeq2 package (version 1.38.0) (13). Volcano plots of the results were drawn through Xiantao (https://xiantaozi.com), a comprehensive web service for biomedical data analysis and visualization.

### 2.5 Weighted gene co-expression network analysis

WGCNA (version 1.71) package was used to identify the gene expression modules most associated with UC. The steps were as followed (1) hierarchical clustering analysis was performed to identify outliers in the sample. (2) the pickSoftThreshold function was utilized to screen out soft-power parameters. (3) a topological overlap matrix (TOM) is created by converting the matrix of correlations with the most appropriate b value to an adjacency matrix and then into a topological overlap matrix. (4) based on the average linkage hierarchical clustering, an hierarchical clustering tree (linked gene best fit) was constructed, and then the dynamic tree cut algorithm (minModuleSize = 30) was used to find different gene modules. (5) gene modules and clinical phenotypes (CON and UC) were correlated using the Pearson correlation coefficient. the modules with correlation coefficient > 0.4 and Pvalue < 0.05 were selected.

### 2.6 Screening of hub genes using machine learning-based integrative approaches

Seven machine-learning algorithms were used to establish a consensus on efferocytosis-related genes, evaluated with three commonly used metrics: AUC (Area Under the Curve), Accuracy, and F1 Score. The integrated algorithms encompassed a range of techniques, including, elastic network (Enet), Least Absolute Shrinkage and Selection Operator (Lasso), Ridge, Support Vector Machine(SVM), Naive Bayes Algorithm (NB), Gradient Boosting Machine (GBM) and eXtreme Gradient Boosting (XGBoost). The procedure involved the following steps: (1) Differentially efferocytosis-related genes were obtained by intersecting genes of efferocytosis geneset, DEGs and mudules geneset. (2) The glmnet package was utilized to perform Elastic Net analysis. A range of alpha (0-1, Step size 0.1) values was tested to find the optimal regularization parameter. Cross-validation was employed to assess model performance, and the results were stored in fit_results. The optimal alpha values were determined based on the minimum lambda values obtained from the cross-validation process. (3) Lasso and Ridge regression was conducted using cv.glmnet with alpha value 1 or 0, and the model was trained using k-fold cross-validation. (4) The caret and e1071 packages were employed for SVM analysis. A radial kernel was used, and the model was trained using repeated cross-validation. (5) Naive Bayes, GBM, and XGBoost classification was performed using the caret package, and the model was trained using repeated cross-validation. (6) caret and pROC packages were used to calculate AUC, Accuracy, and F1 Score of each model. (7) Select the models with the highest score among the three evaluation metrics, and extract the intersection of their features as hub genes. For models that cannot perform feature selection, such as SVM and XGBoost, feature selection is carried out by integrating RFE (Recursive Feature Elimination).

### 2.7 Molecular docking analysis

GRAMM was used to perform molecular docking of the proteins encoding by hub genes with UST. GRAMM (Graphical Representation of Molecular Mechanics) docking is a computational method used for molecular docking, particularly suitable for studying the interactions between large molecules, such as proteins. The 3D coordinates of UST (PDB ID: 3HMW) and hub proteins (ANXA1:1MCX; PANX1:6M02; ANXA5: 1avr; CD93:8A59; SERPINE: 1A7C; MGFE8: AF-Q08431-F1-v4) were downloaded from The Protein Data Bank (http://www.rcsb.org) and The AlphaFold Protein Structure Database (https://alphafold.ebi.ac.uk/). For the docking analysis, all protein files were converted to the PDBQT format, with all water molecules excluded and polar hydrogen atoms added, the docking methodology selected is free docking, with all other parameters set to their default values. In the 10 models output by GRAM, the best model is selected for input into PDBePISA (https://www.ebi.ac.uk/msd-srv/prot_int/pistart.html) to perform binding energy calculations. The molecular docking visualization is completed using pymol.

### 2.8 Biological function and pathway enrichment analysis

Kyoto Encyclopedia of Genes and Genomes (KEGG) pathway enrichment analysis and GSEA (Gene Set Enrichment Analysis) was performed by the ClusterProfiler (4.7.1.003) package.

### 2.9 Hub genes validation in mice transcriptome data

The raw data of colitis mice was retrieved from the GEO database under accession number GSE227407. After preprocessing, including quality control and normalization, we performed differential expression analysis using DESeq2. Then, the homologene(version 1.4.68.19.3.27) package was used to convert gene symbols from human to mouse. This process is crucial for ensuring the consistency of gene nomenclature across species. Subsequently, we identified the intersection of differentially expressed genes in mouse colon tissue with the hub genes, which were determined through a consensus approach among various machine learning models.

### 2.10 Animal model of colitis

Male C57BL/6J mice (~25g) were obtained from Gempharmatech (Chengdu, China). This study was performed in accordance with the recommendations in the Guide for the Care and Use of laboratory Animals of the National Institutes of Health. The protocol was approved by the Committee on the Ethics of Animal Experiments of Chengdu University of Traditional Chinese medicine. Mice were randomized into Control group and DSS group. Mice in DSS group was provided 2.5% DSS (MP Biomedicals, Santa Ana, CA, USA) in the drinking water for 7 days, and the Control group was only administered distilled water.

### 2.11 Hub genes validation in mice by ASTRAL-DIA proteomic data

Colon tissue proteins from DSS-induced colitis mice (n=6/group) were extracted, digested, and analyzed by liquid chromatography-mass spectrometry (LC-MS). Differentially abundant proteins were identified with statistical significance (P<0.05, FC>1.2). Instrument parameters and processing details in **Supplementary Data Sheet 1**.

### 2.12 Statistical analysis

SPSS 26.0 software was used for statistical analysis, and the measurement data were expressed as Mean ± SD. Perform normality tests and tests for homogeneity of variance prior to statistical analysis. For comparisons between two independent sample groups, if the data met the criteria for normal distribution and homogeneity of variance, we used a two-independent-samples t-test. If the data were normally distributed but had unequal variances, we employed the more robust Welch test. If the data did not meet the criteria for normal distribution and homogeneity of variance, we used a non-parametric test—the Wilcoxon rank-sum test. P<0.05 indicates statistically significant difference. Pictures were drawn with GraphPad Prism software (version 8.0).

## 3 Results

### 3.1 Single-cell sequencing reveals the activation of efferocytosis in different cell types during colitis

**Figure 1:**
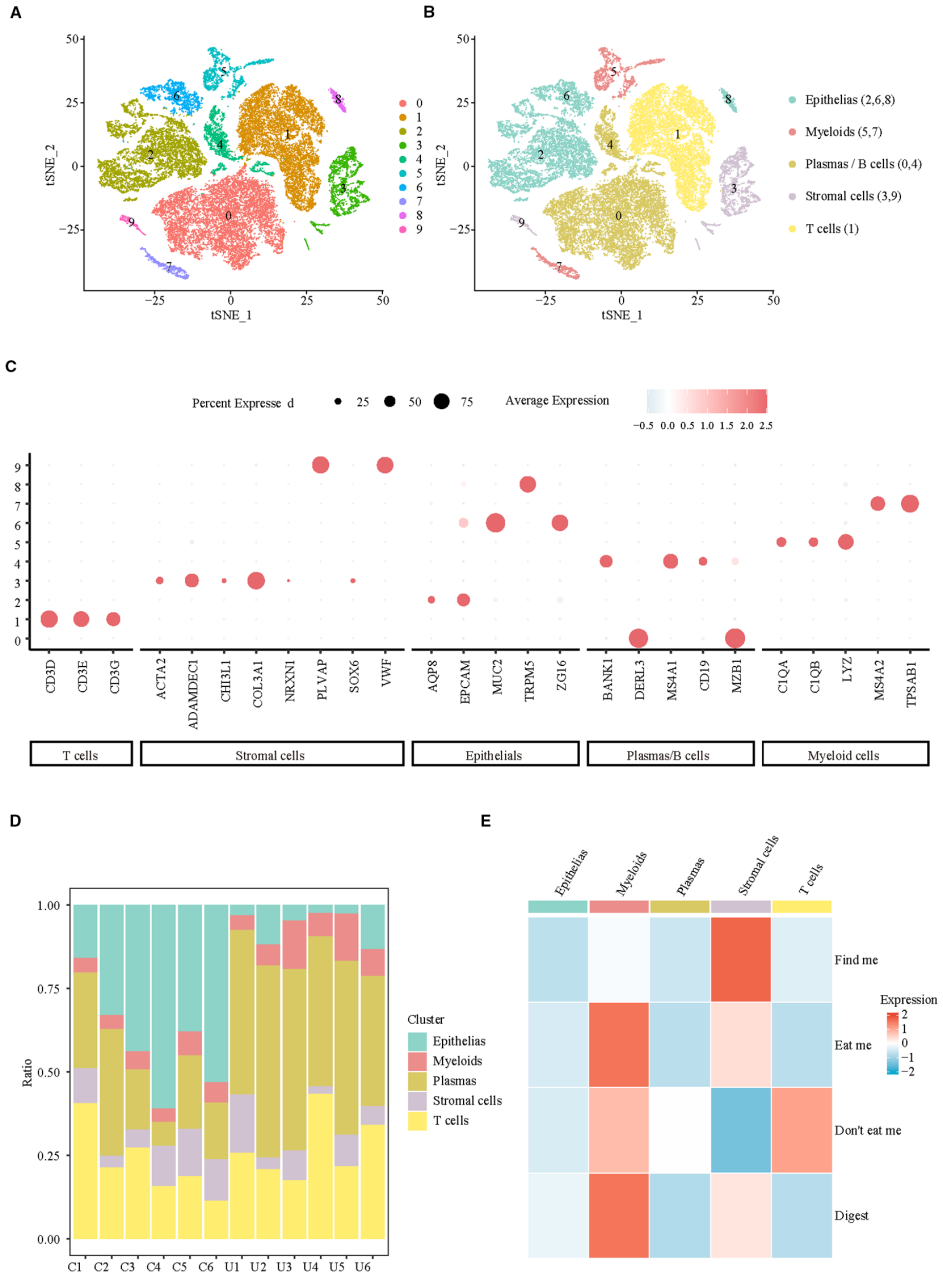
Integrating single-cell analyses from healthy and UC samples reveals the cell specificity of efferocytosis signaling. **(A)** UMAP representation of scRNA-seq data for the clusters. **(B)** UMAP showing annotation of major cell types identified by scRNA-seq. **(C)** Dotplot of top marker genes discriminating the different cell subsets. **(D)** Barplots representing the proportions of each cell type resolved by scRNA-seq for CON and UC. **(E)** Heatmap of efferocytosis scores in different cell types.

To clarify the changes in colon efferocytosis signals at the cellular level in UC patients, a total of 37,433 cells from colonic biopsies of CON (n=6) and UC (n=6) active patients were included in the analysis. Subsequently, we filtered out the low-quality cells, resulting in 32,972 cells that were identified as belonging to 10 clusters (**Figure 1A**, **Supplementary Figure S1**) and 5 major cell populations (**Figure 1B**). The Dotplot was utilized to illustrate the expression of published markers within cell clusters (12), thereby substantiating the reliability of cell type identification (**Figure 1C**). The analysis of cellular proportions in various samples revealed that, compared to the control group, patients with ulcerative colitis (UC) exhibited a significant decrease in the proportion of colonic epithelial cells, along with a notable increase in the levels of myeloid cells and Plasma/B cells (**Figure 1D**). Analysis of efferocytosis signaling indicates that myeloid cells exhibit the strongest eat-me and digest signals, while the ‘find me’ chemotactic function, which is crucial for efferocytosis chemotaxis, is predominantly activated in stromal cells (**Figure 1E**). These findings suggest that the enhancement of efferocytosis signaling in ulcerative colitis (UC) is primarily associated with myeloid and stromal cells.

### 3.2 Single-cell sequencing reveals the heterogeneity in efferocytosis signaling among different macrophage subtypes

**Figure 2:**
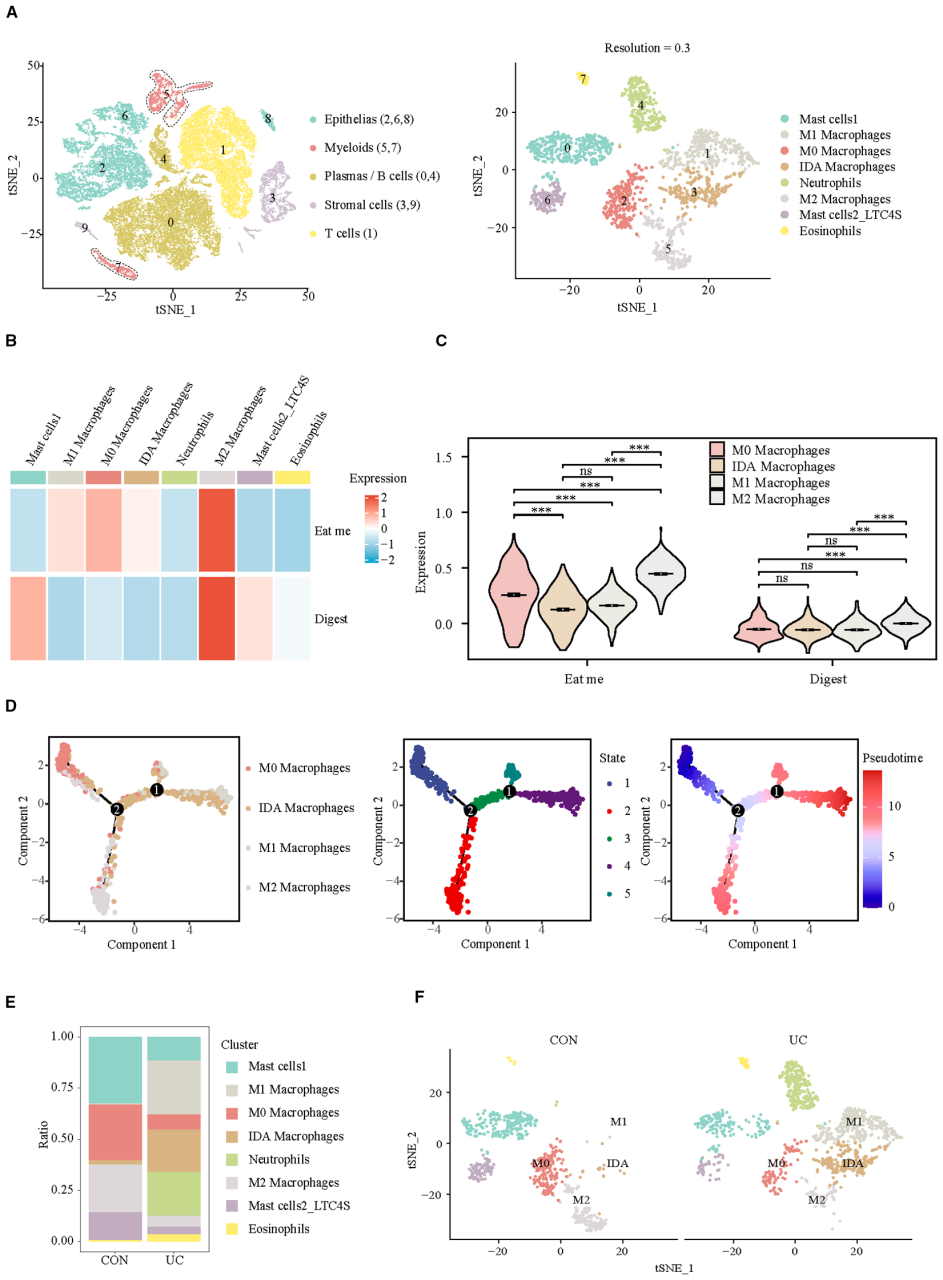
Analysis of efferocytosis in myeloid cell subsets. **(A)** UMAP representation of the detailed subpopulations of myeloid cells. **(B)** Heatmap of efferocytosis scores in subpopulations of myeloid cells. **(C)** Violin plot of efferocytosis scores of different macrophage subpopulations. **(D)** Trajectory analysis reveals the differentiation of different macrophage subpopulations into two distinct directions (M1/IDA or M2). **(E)** Barplots representing the proportions of subpopulations within myeloid cells. **(F)** UMAP visualization of myeloid cell subpopulations across CON and UC group. ***P< 0.001, ns, no significance.

Considering the crucial role of myeloid cells in eat me and digest signals, we identified their subpopulation composition. At a resolution of 0.3, eight clusters were identified, which were categorized into four subpopulations: mast cells (cluster: 0, 6), macrophages (cluster: 1, 2, 3, 5), neutrophils (cluster: 4), and eosinophils (cluster: 7) (**Figure 2A**, **Supplementary Figure S2**). The analysis of efferocytosis across various cell subtypes indicates that macrophages, particularly the M2 subtype, exhibit the strongest efferocytosis capabilities (**Figures 2B, C**). Pseudotime analysis indicates that the efferocytosis capability of macrophages is inversely related to their pro-inflammatory phenotype. After differentiation into the M2 subtype, macrophages exhibit the strongest efferocytosis ability, whereas differentiation into the M1 subtype results in the weakest efferocytosis capability (**Figure 2D**). However, in the colon of patients with active ulcerative colitis (UC), the proportion of M0 and M2 macrophages is significantly decreased, while the proportion of M1 and IDA macrophages is markedly increased (**Figures 2E, F**). Our results confirm M2 macrophage depletion and concomitant efferocytosis impairment in UC patients, consistent with the established anti-inflammatory role of M2 macrophages in inflammation resolution (14)—yet direct causality warrants future experimental validation.

**Figure 3:**
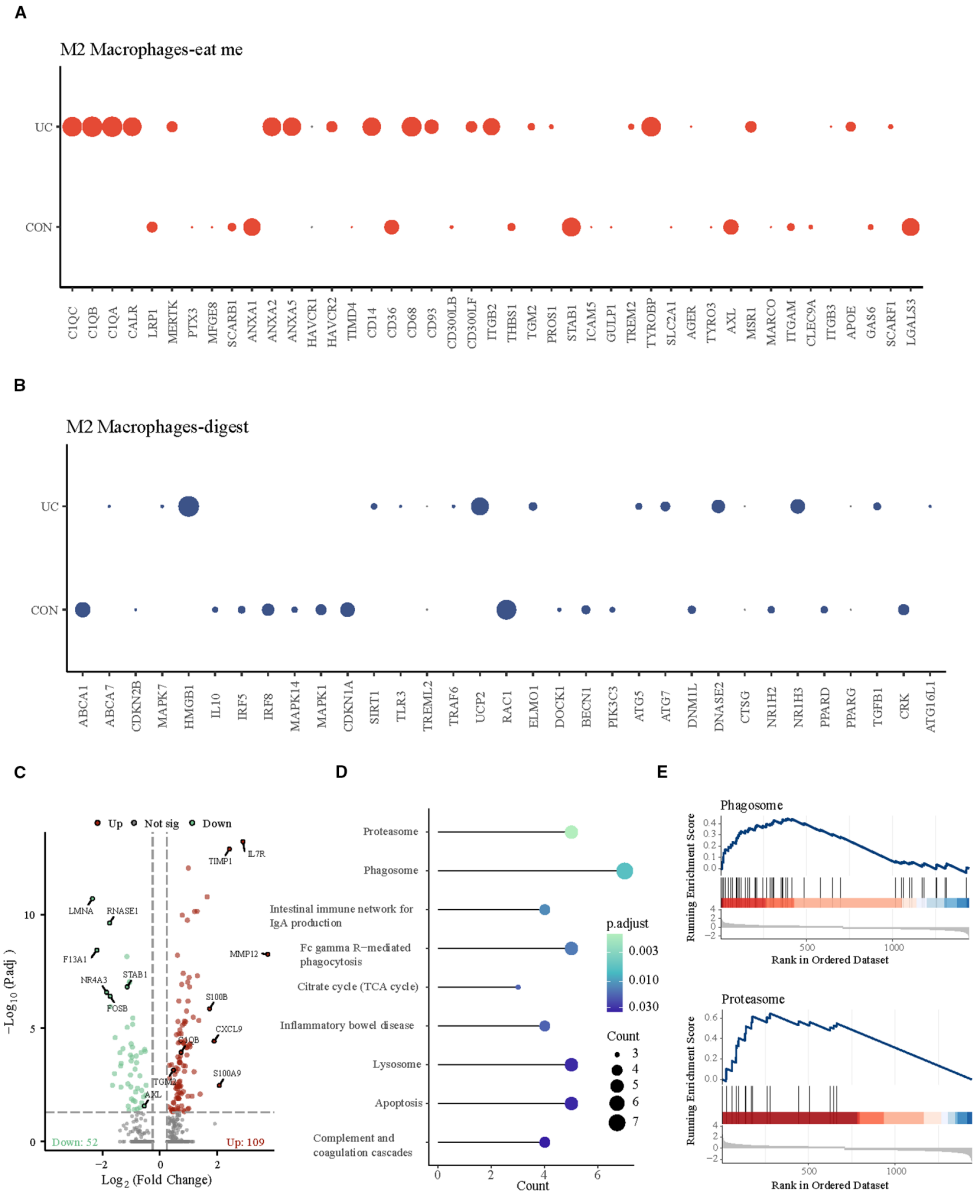
Analysis of M2 macrophage efferocytosis signaling between CON and UC group. **(A)** Dotplot representation of the expression abundance of chemotactic (eat-me) molecules in M2 macrophage subpopulations across groups. **(B)** Dotplot representation of the expression abundance of digest molecules in M2 macrophage subpopulations across groups. **(C)** Volcano plot representation of differential gene expression of M2 macrophage subpopulations between CON and UC group. **(D)** Stick plot displays the KEGG enrichment pathways of differential genes in M2 subpopulations. **(D)** Gene set enriched in the phagosome pathway (NES = 2.27, FDR = 0.005189). **(E)** Gene set enriched in the proteasome pathway (NES = 2.35, FDR = 0.00647092).

Subsequently, we observed the differences of efferocytosis signals in M2 macrophages between CON and UC group. Compared with the CON group, M2 macrophages in UC group exhibited increased expression of eat me molecules, suggesting that inflammatory stimuli may enhance the phagocytosis capability of M2 macrophages (**Figures 3A, B**). To verify this hypothesis, we conducted enrichment analysis on the differentially expressed genes between the macrophage subtypes. The results demonstrated significant enrichment of upregulated genes in the Proteasome and Phagosome (**Figures 3C–E**), suggesting that M2 macrophages may play a more active role in the colon of patients with active ulcerative colitis. By enhancing proteasome and phagosome functions, they can more effectively clear apoptotic cells, which may contribute to reducing inflammation.

### 3.3 Single-cell sequencing reveals the homogeneity in find me signaling among different fibroblast subtypes

**Figure 4:**
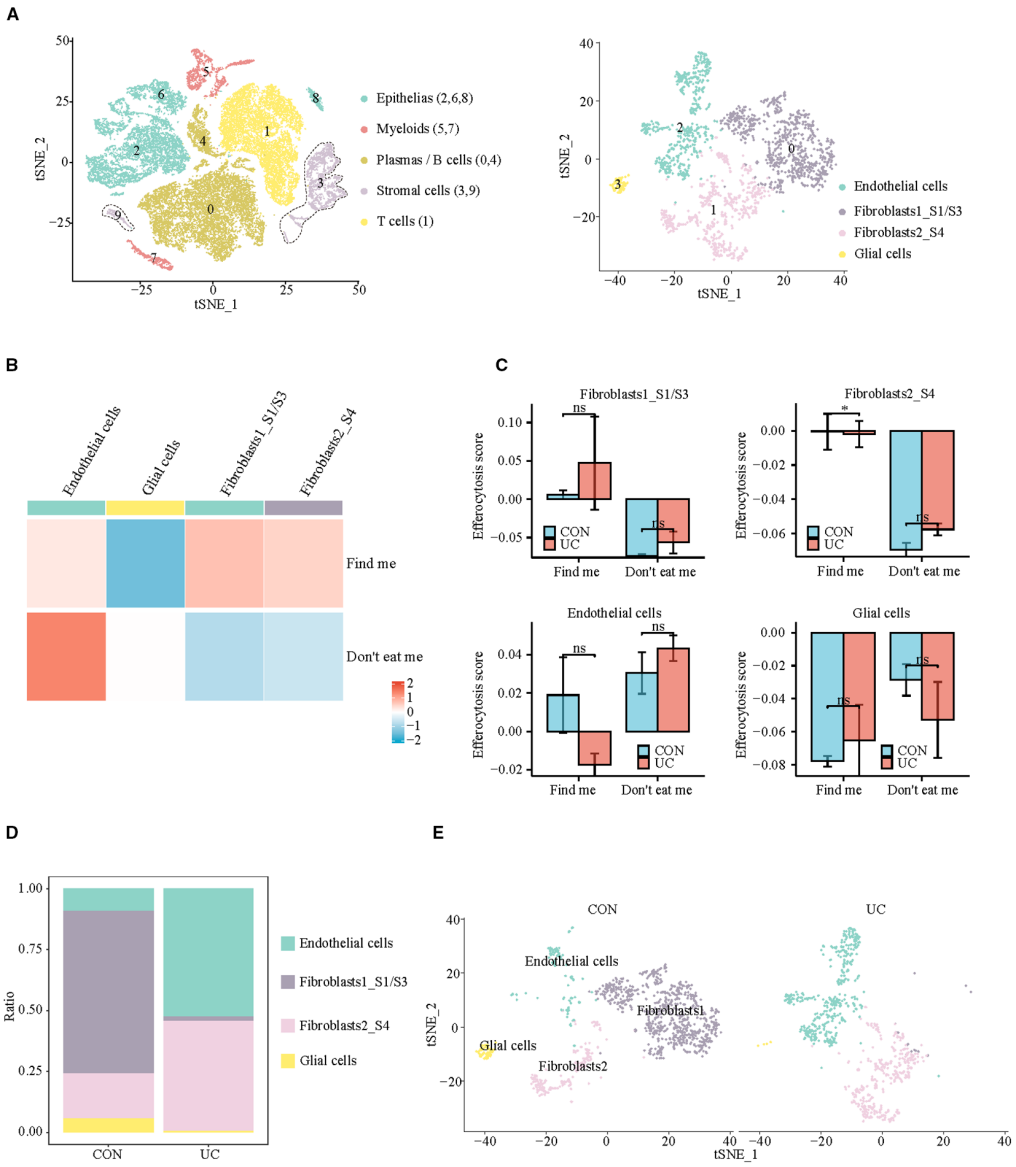
Analysis of efferocytosis in stromal cell subsets. **(A)** UMAP representation of the detailed subpopulations of stromal cells. **(B)** Heatmap of efferocytosis scores in subpopulations of stromal cells. **(C)** Barplot of efferocytosis scores in subpopulations of stromal cells across groups. **(D)** Barplot representing the proportions of subpopulations within stromal cells. **(E)** UMAP visualization of stromal cell subpopulations across CON and UC group.

Subsequently, we analyzed the stromal cells with the strongest chemotactic, find me signals. At a resolution of 0.1, four clusters were identified, which were categorized into 3 subpopulations: Endothelial cells (cluster: 2), Fibroblasts (cluster: 0, 1), and Glial cells (cluster: 3) (**Figure 4A**, **Supplementary Figure S3**). In which Fibroblast1_S1/S3 (cluster 0) is primarily responsible for the production and maintenance of the extracellular matrix, while Fibroblast2_S4 (cluster 1) is involved in inflammation and collagen cross-linking (12, 15). The analysis of efferocytosis across various cell subtypes indicates that fibroblasts and endothelial cells have similar chemotactic abilities (**Figure 4B**). The intergroup comparison among the same type of cells shows that, apart from a slight decrease in the chemotactic ability of inflammatory fibroblasts (Fibroblast2_S4) in UC patients, there are no significant differences between other cell subtypes across the groups (**Figure 4C**). Quantitatively, the proportion of fibroblasts_S1/3, with higher chemotactic ability, significantly decreases in UC, while inflammatory fibroblasts_S4 become the predominant type of fibroblast in UC (**Figures 4D, E**).

### 3.4 Cell communication analysis reveals receptor-ligand genes of efferocytosis in the interaction between fibroblasts and macrophages

**Figure 5:**
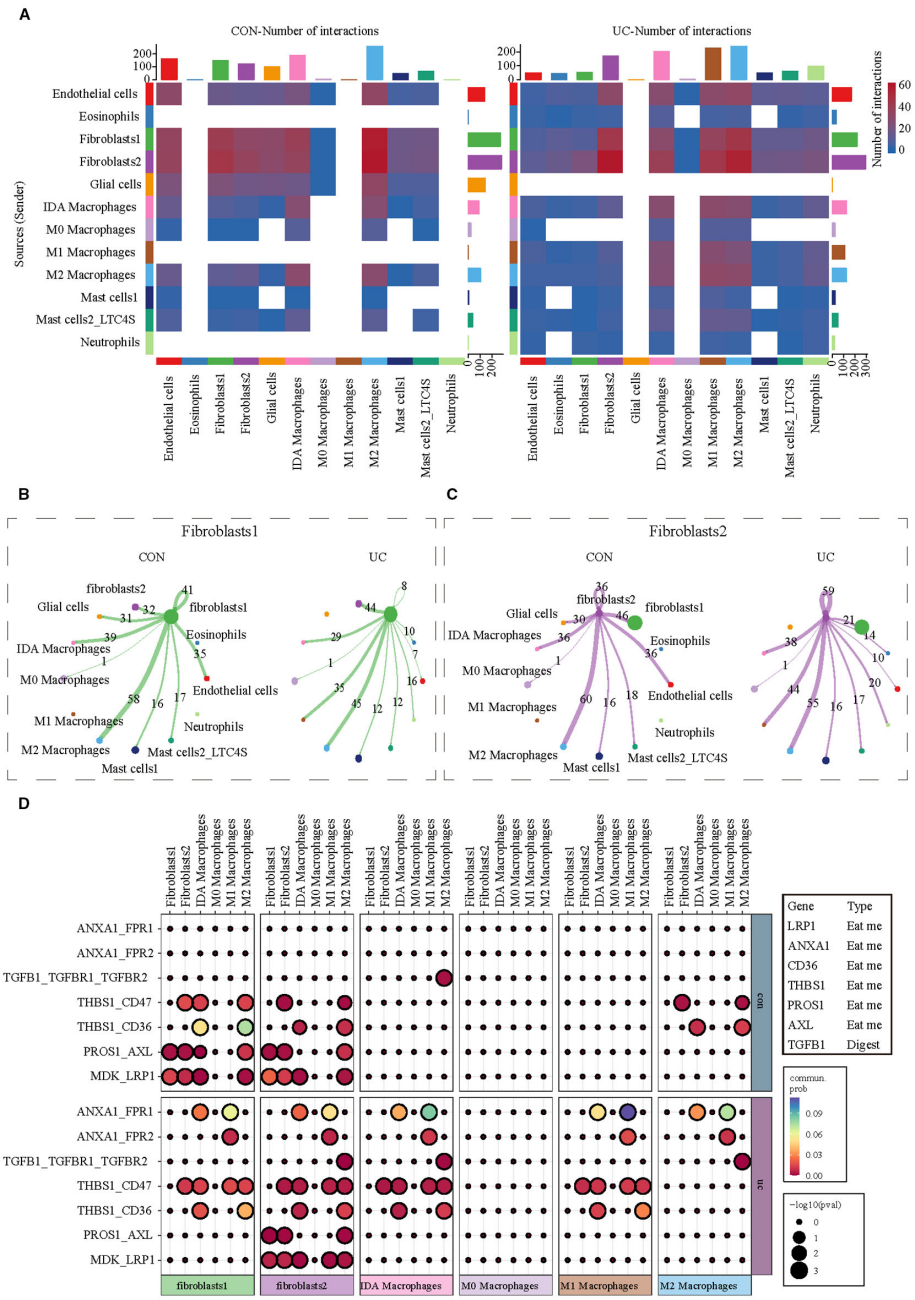
Analysis of cell communication between myeloid cells and stromal cells. **(A)** CellChat analysis indicating alterations in cell communication between stromal cells and myeloid cells between CON and UC colon tissues. **(B)** The communication network between Fibroblast 1 and myeloid cell subtypes. **(C)** The communication network between Fibroblast 2 and myeloid cell subtypes. **(D)** The heatmap illustrates the changes in efferocytosis-related ligand-receptor signals between fibroblasts and macrophages across different groups.

Given the distinct activation characteristics of efferocytosis signals in fibroblasts and macrophages, we conducted cell communication analysis to observe whether UC enhances the interaction between fibroblasts and macrophages in the colon, as well as their potential interaction molecules. Consistent with our expectations, the communication analysis of myeloid cells (the cell group to which macrophages belong) and stromal cells (the cell group to which fibroblasts belong) shows that, regardless of whether in the CON or UC group, fibroblasts are the cell type with the most interactions with macrophages. M1 macrophages are the cell type with the most significant changes in interaction numbers in the UC colon (**Figure 5A**). The separate analysis of fibroblasts indicates that, compared to the CON group, the number of interactions between colonic fibroblast 1 and M1 macrophages in the UC group increased from 0 to 35 pairs, while the number of interactions with M2 macrophages decreased from 58 to 45 pairs (**Figure 5B**). For fibroblast 2, the number of interactions with M1 macrophages increased from 0 to 44 pairs, and the number of interactions with M2 macrophages decreased from 60 to 55 pairs (**Figure 5C**). The analysis of efferocytosis-related ligand-receptor pairs indicates that the efferocytosis interactions between the two types of fibroblasts and macrophages are mediated by seven ligand-receptor pairs, with significant heterogeneity. Compared to the CON group, the efferocytosis interactions between the two types of fibroblasts and M1 macrophages in the UC colon are enhanced, primarily mediated by the ligand-receptor pairs ANXA1_FPR1, ANXA1_FPR2, and THBS1_CD47. However, the number of efferocytosis ligand-receptor pairs between fibroblast 1 and IDA macrophages and M2 macrophages decreases (the interactions PROS1_AXL and MDK_LRP1 disappear), while the number of efferocytosis ligand-receptor pairs between fibroblast 2 and IDA macrophages (ANXA1_FPR1 and THBS1_CD47) and M2 macrophages (TGFB1_TGFBR1_TGFBR2) significantly increases (**Figure 5D**).

### 3.5 Identifying key efferocytosis-related genes associated with UC based on bulk RNA-seq

**Figure 6:**
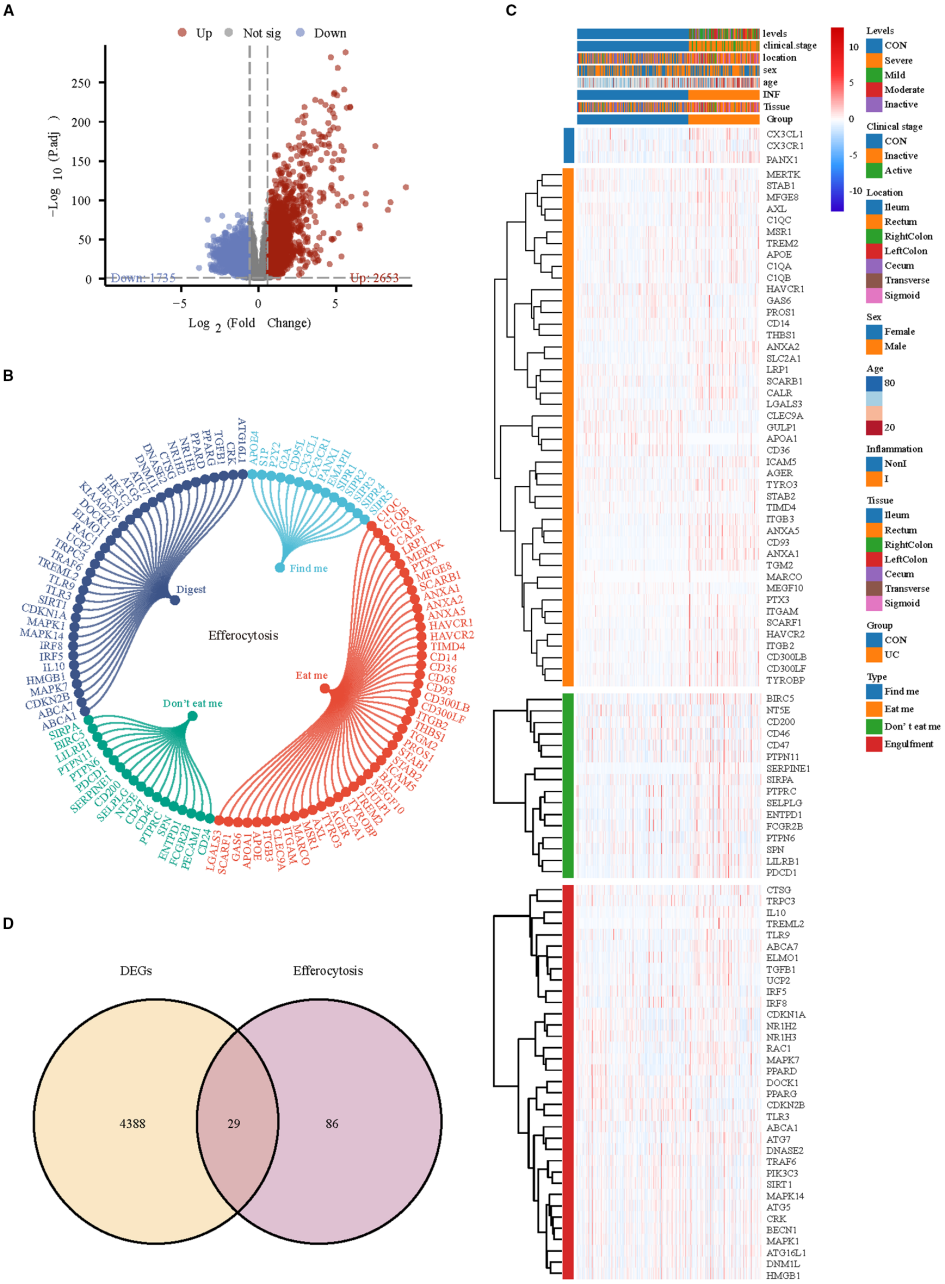
Differential efferocytosis gene screening in bulk RNA-seq data. **(A)** Volcano plot representation of differential gene expression in GSE193677. **(B)** The efferocytosis gene list contains 115 genes, of which 14 are from findme, 47 are from eatme, 18 are from Don’t eat me and 36 digest gene. **(C)** Heatmap displays the expression trends of detectable efferocytosis genes. **(D)** Venn diagram shows the intersection of differential genes with efferocytosis-related genes.

Subsequently, we analyzed bulk RNA-seq data from large-scale UC public datasets to validate the discoveries identified in the scRNA-seq dataset. The sets GSE193677 were normalized, and the gene expression with biological significance was obtained. There were 4388 genes differentially expressed between 293 UC samples and 461 healthy control samples, including 2653 up-regulated genes and 1735 down-regulated genes (**Figure 6A**). Of the 115 efferocytosis-associated genes identified, expression was detected for 99 within the dataset (**Figures 6B, C**). To obtain reliable differential efferocytosis-related genes, we screened the intersection of differential genes and efferocytosis-related genes based on the standard of |Fold Change| ≥ 1.5. A total of 29 differential efferocytosis-related genes were screened, 24 were up-regulated, and 5 were down-regulated (**Figure 6D**).

**Figure 7:**
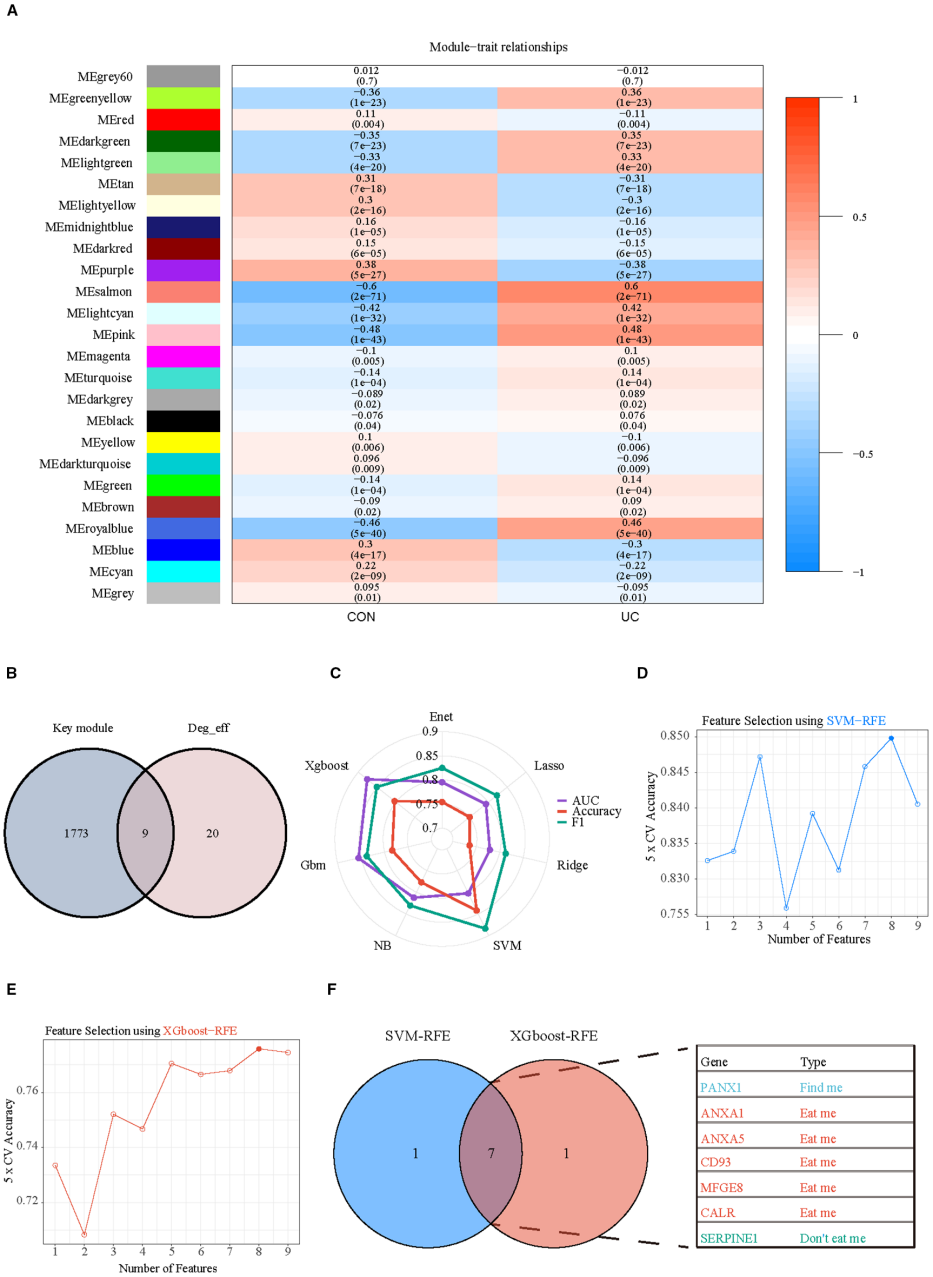
Selection of key hub efferocytosis genes. **(A)** Heatmap displays the correlation between module genes and disease in WGCNA analysis. Correlations and adjusted pvalue are included in each cell. **(B)** Venn diagrams illustrate the intersection of key module genes from WGCNA with differentially expressed efferocytosis genes. **(C)** Radar chart represents the performance of seven machine learning models evaluated using three performance metrics. **(D)** SVM-RFE (Support Vector Machine Recursive Feature Elimination) analysis for screening hub genes. Type accuracy using fivefold cross-validation of different feature combination models. **(E)** XGboost-RFE (eXtreme Gradient Boosting Recursive Feature Elimination) analysis for screening hub genes. Type accuracy using fivefold cross-validation of different feature combination models. **(F)** Venn diagrams illustrate the intersection of the optimal feature genes selected by SVM-RFE and XGboost-RFE.

Subsequently, WGCNA with fit index ≥ 0.85 and β= 7 was applied to further screen the differential efferocytosis-related genes associated with UC (**Supplementary Figures S4A–C**). In a total of 25 co-expression modules, the Pink, Salmon, RoyalBlue, and LightCyan modules showed a strong positive correlation with UC (**Figure 7A**). By intersecting the genes from the key modules with the differential efferocytosis-related genes, a total of 9 characteristic genes were obtained (**Figure 7B**). Then, we developed a machine learning framework designed to identify hub genes by addressing seven distinct binary classification tasks. Through systematic evaluation of algorithm performance metrics (AUC, accuracy, and F1-score), our comparative analysis revealed that support vector machine (SVM) and extreme gradient boosting (XGBoost) exhibited superior predictive efficacy compared to other models (**Figure 7C**). By integrating Recursive Feature Elimination (RFE) for feature selection, we identified seven hub genes (Panx1, Anxa1, Anxa5, Cd93, Mfge8, Calr, and Serpine1) that were consistently prioritized by both algorithms (**Figures 7D–F**).

### 3.6 Multiple datasets confirmed that the expression of hub genes is associated with the severity of UC

**Figure 8:**
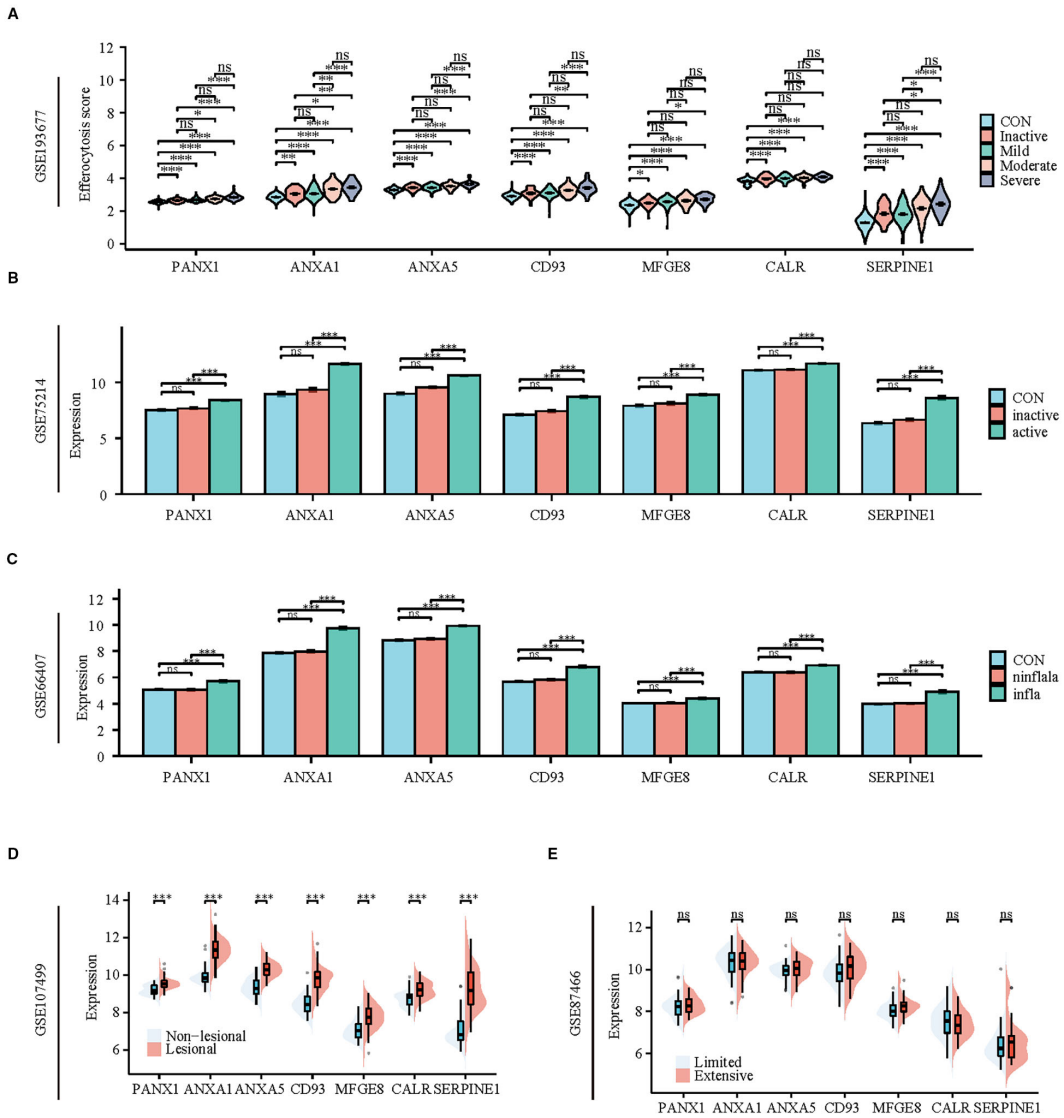
Patients with active UC and inflammatory tissues of the colon exhibit high expression of hub genes and TFs of efferocytosis. **(A)** Violin plot display the expression of hub genes between healthy individuals and patients with varying degrees of severity. **(B)** Bar charts display the expression of hub genes between healthy individuals and patients in active and inactive stage. **(C)** Bar charts display the expression of hub genes among healthy colons, inflammatory colons, and non-inflammatory colons. **(D)** Split violin plot revealing the expression differences in hub genes between lesional tissues and non-lesional tissues. **(E)** Split violin plot revealing the expression differences in hub genes between limited colitis and extensive colitis. *P < 0.05, **P < 0.01, ***P < 0.001, ns, not significant.

To validate the reliability of the hub genes, we verified their expression in multiple datasets (GSE193677, GSE75214, GSE66407, GSE107499, GSE87466) that encompass various disease stages and different injury conditions. In the dataset GSE193677, the expression of hub genes in colonic tissue increases with the severity of the disease. Except for MFGE8 and CALR, the expression of other hub genes in severe patients is significantly higher than in patients with mild or lower degrees of the disease or in healthy individuals (**Figure 8A**). Similarly, in the dataset GSE75214, the expression of hub genes in active phase colonic tissue is significantly higher than in healthy and inactive phase tissues (**Figure 8B**). Furthermore, datasets GSE66407 and GSE107499 show that there is no difference in the expression of hub genes between healthy and non-inflammatory/non-lesional tissues, with high expression primarily in inflammatory or lesional tissues (**Figures 8C, D**). Additionally, we found that the expression of hub genes is not related to the extent of UC lesions, as there is no difference in their expression between extensive and limited tissues (**Figure 8E**).

### 3.7 Hub genes of efferocytosis are associated with UC patients’ response to ustekinumab

**Figure 9:**
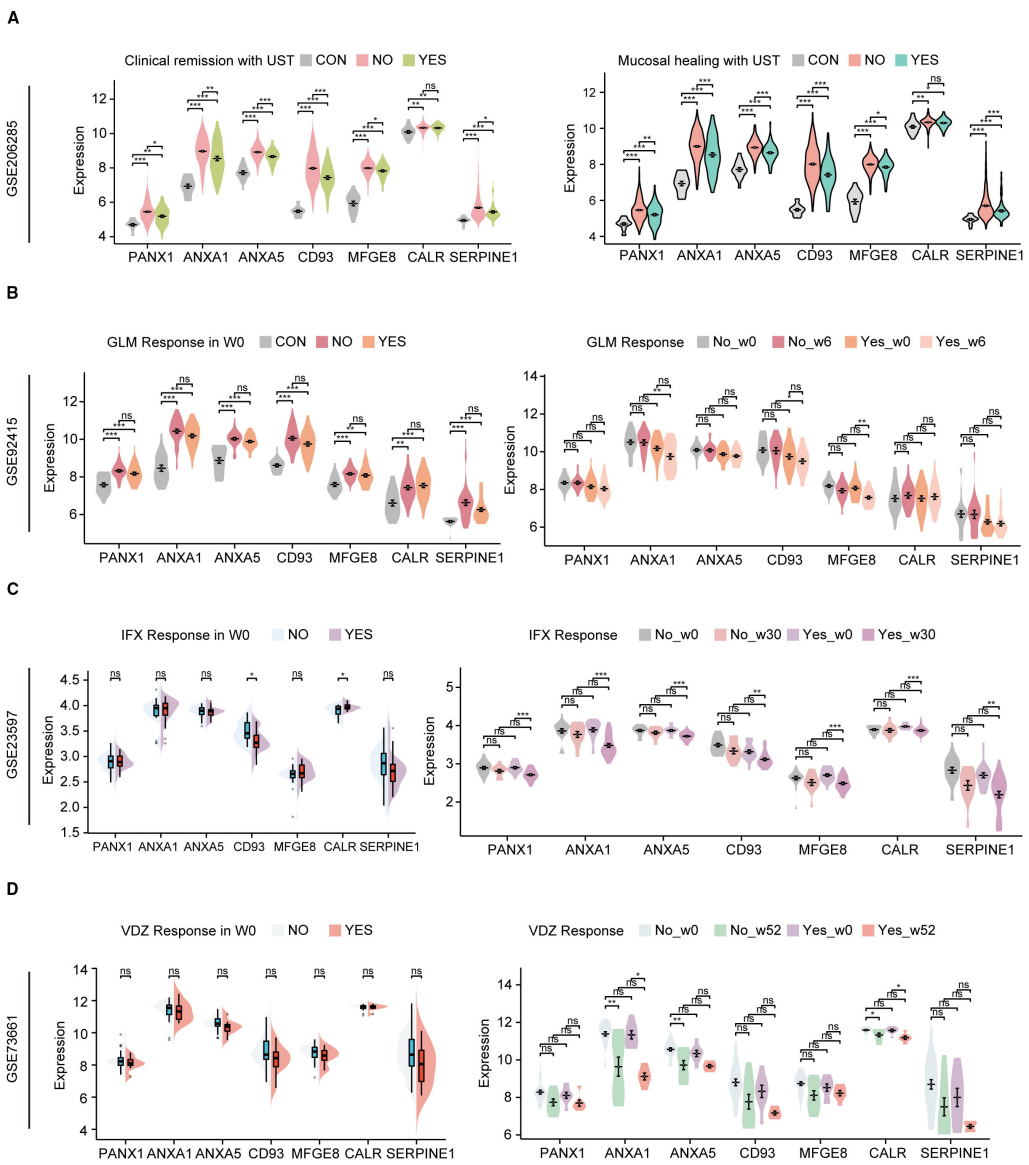
Hub genes of efferocytosis are associated with UC patients’ response to biologics. **(A)** The relative expression levels of hub genes in the colonic mucosa of healthy controls, UC patients in not responding and responding groups before UST therapy in GSE206285 **(B)** The relative expression levels of hubs in the colonic mucosal of healthy controls, UC patients in responding and non-responding groups before and after GLM treatment in GSE92415. **(C)** The relative expression levels of hubs in the colonic mucosal of healthy controls, UC patients in responding and non-responding groups before and after IFX treatment in GSE23597. **(D)** The relative expression levels of hub genes in the colonic mucosa of healthy controls, UC patients in not responding and responding groups before and after VDZ therapy in GSE73661. UST, ustekinumab; GLM, golimumab; IFX, infliximab; VDZ, vedolizumab. *P < 0.05, **P< 0.01, ***P < 0.001, ns, not significant.

Recently, biologics such as UST, Vedolizumab (VDZ, Anti-α4β7) and TNF-a inhibitors Infliximab (IFX) and Golimumab (GLM) have been approved for treating ulcerative colitis and proposed as first-line agents (16). Still, some patients may not respond to biologics or respond poorly. The effects of biologics on hubs were explored using the GSE206285, GSE92415, GSE23597 and GSE73661. GSE206285 contains expression profiles of biopsy samples from UC patients treated with UST. Before UST treatment, apart from CALR, the expression of hub genes in non-responsive UC patients is significantly higher than in healthy individuals and patients who respond to treatment, while the expression in responsive patients is higher than in healthy individuals, regardless of whether the evaluation is based on clinical remission or mucosal healing (**Figure 9A**). ROC analysis further confirmed the predictive capacity of these hub genes, with the multi-gene model achieving AUCs of 0.671 for clinical remission and 0.693 for mucosal healing (**Supplementary Figure S5**). However, except for UST, no differences in the expression of hub genes were observed between responsive and non-responsive patients before the administration of GLM, IFX, and VDZ, although some genes showed downregulation in the responsive group after the medication was given (**Figures 9B–D**). To investigate the potential interaction between UST with the key hub genes except CALR, molecular docking studies were performed. With the exception of ANXA5 (**Supplementary Figure S6C**), all other hub genes are capable of forming structures with the light or heavy chains of UST with binding energies lower than -5 kcal/mol (**Supplementary Figures S6A, B, D–F**), suggesting that the proteins encoded by these genes have a strong affinity for UST and may form stable complexes. These results suggest that efferocytosis signaling is a key factor affecting drug responsiveness in UST.

### 3.8 Animal models validate the hub genes of efferocytosis signaling

**Figure 10:**
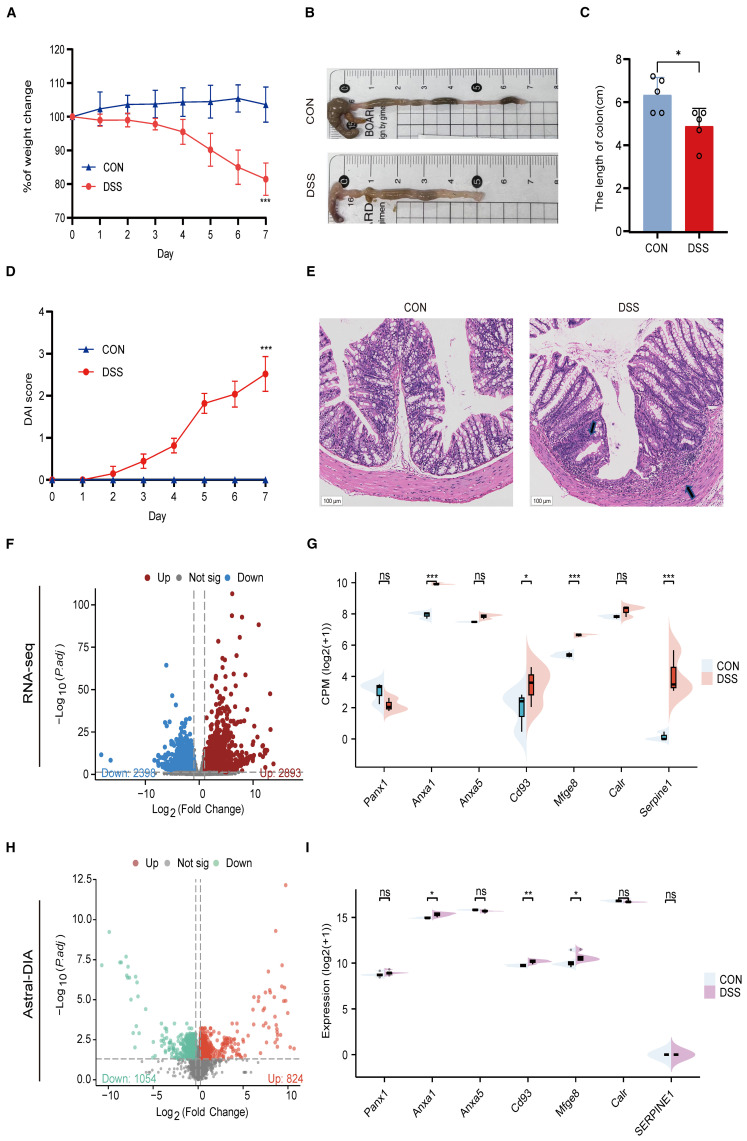
Validation of hub genes in a DSS-induced colitis model. **(A)** Body weight changes between CON and UC mice. **(B)** Representative images of colons between CON and UC mice. **(C)** Colonic length changes between CON and UC mice. **(D)** DAI score changes between CON and UC mice. **(E)** Representative images of HE staining in the colon tissues (magnification ×100) and arrows indicate inflammatory infiltrates. **(F)** Volcano plot representing differential gene expression between CON and DSS mice. **(G)** Split violin plot revealing the expression differences in hub genes between CON and DSS mice. **(H)** Volcano plot representing differential protein expression between CON and DSS mice. **(I)** Split violin plot revealing the expression differences in hub proteins between CON and DSS mice. Data are shown as mean ± SD. *P<0.05, **P<0.01, ***P<0.001, ns, not significant.

To further validate the important role of hub genes in UC, we employed the DSS induced mouse colitis model. Compared with the CON group, mice in DSS group showed obvious weight loss (**Figure 10A**), decreased colon length (**Figures 10B, C**), increased DAI score (**Figure 10D**) and severe inflammatory infiltrates (**Figure 10E**).

Then, we detected the expression levels of hub genes using RNA-seq and ASTRAL-DIA Proteomic (**Figures 10F, H**). Compared to the control group, the mRNA and protein levels of Anxa1, Cd93, and Mfge8 were significantly upregulated in the DSS group, while other hub genes did not show a consistent trend with clinical data (**Figures 10G, I**). These results provide cross-species evidence supporting the function and regulation of efferocytosis signaling in UC.

## 4 Discussion

Efferocytosis, also known as apoptotic cell clearance or programmed cell removal, is a specialized form of phagocytosis that plays a pivotal role in maintaining tissue homeostasis, mitigating post-injury inflammatory responses, and facilitating tissue regeneration (17). Emerging evidence highlights its regulatory function in modulating inflammatory cascades, positioning efferocytosis as a promising therapeutic target for colitis management (5, 18). This therapeutic strategy shows considerable promise as a future direction for the treatment of the disease in ongoing research. Nonetheless, the pathological implications of efferocytosis in the context of ulcerative colitis are not yet fully elucidated. Consequently, deepening our understanding of efferocytosis and pinpointing novel therapeutic targets for UC is of critical importance.

Efferocytosis is a multistep process encompassing distinct phases, including “find me,” “eat me,” and “digest me.” Our multi-omics investigation reveals that efferocytosis—a coordinated process involving myeloid and stromal cells—plays a pivotal role in UC pathogenesis. Myeloid cells, particularly M2-polarized macrophages, dominate apoptotic cell clearance via upregulated “eat me” (e.g., ANXA1, CD93) and “digest me” pathways (proteasome and phagosome activation), while stromal cells orchestrate “find me” chemotactic signals. This spatial coordination highlights myeloid-stromal crosstalk as a key driver of efferocytosis in UC.

Macrophages are the primary cell type executing efferocytosis, playing a crucial role in clearing damaged cells and suppressing inflammatory responses during UC (19, 20). Functionally polarized macrophages are classified into pro-inflammatory M1 and anti-inflammatory M2 subtypes (21, 22). In our study, analysis of single-cell datasets from UC patients revealed an inverse correlation between macrophage efferocytic capacity and pro-inflammatory phenotypes: M2-polarized macrophages exhibited the highest efferocytic activity, whereas M1-polarized macrophages displayed the lowest. Notably, M2 macrophages in UC colonic tissues showed upregulated expression of “eat me” molecules, suggesting inflammation-induced enhancement of their phagocytic potential. Differential gene analysis across macrophage subsets further demonstrated significant upregulation of proteasome- and phagosome-related pathways in M2 macrophages, indicating that enhanced proteasomal and phagolysosomal functions in M2 macrophages facilitate efficient clearance of apoptotic debris, thereby attenuating UC-associated inflammation. Paradoxically, despite enhanced phagocytic machinery in residual M2 macrophages, their proportion is significantly reduced in active UC, suggesting an “efferocytosis exhaustion” mechanism where chronic inflammation depletes anti-inflammatory macrophages, perpetuating tissue damage. This aligns with prior studies linking IL-10 deficiency to impaired efferocytosis (20), though our proteomic data further implicate proteasomal dysfunction as a bottleneck in completing apoptotic clearance.

The identification of six hub genes through machine learning provides novel insights into UC heterogeneity. Through the establishment of ulcerative colitis animal models combined with RNA-seq and ASTRAL-DIA proteomic analyses, we validated that specific hub genes, including Anxa1, Cd93, and Mfge8, exhibited consistent alterations at both the transcriptional and protein levels. ​​Notably, transcriptional discrepancies were observed for PANX1, ANXA5, and CALR in the murine model. We posit that this may be attributed to the inherent limitations of the acute DSS model in fully recapitulating the chronic immune dysregulation and complex tissue remodeling of human UC, and/or to the presence of robust species-specific post-transcriptional regulation (e.g., by microRNAs). This hypothesis is strongly supported by our high-resolution proteomic data, which revealed a critical layer of regulation, as evidenced by the significant dysregulation of SERPINE1 protein despite unchanged mRNA levels. This multi-omics approach underscores that the biological relevance of a hub gene is not solely determined by mRNA abundance and highlights the necessity of protein-level validation to bridge translational gaps. Ultimately, these hub genes exhibit conserved dysregulation across species and correlate with disease severity independent of lesion location. Their biological roles—ANXA1 mediating FPR receptor signaling, CD93 modulating complement cascades, and MFGE8 bridging apoptotic cells to phagocytes—suggest a unified network regulating efferocytosis, further solidifying their importance in UC pathogenesis.

Currently, biological agents including UST (anti-IL-12/IL-23), VDZ (anti-α4β7), and TNF-α inhibitors such as IFX and GLM have been approved for the clinical treatment of ulcerative colitis (16, 23). However, patient responses to these biologics vary significantly, necessitating personalized treatment strategies based on individual drug responsiveness to optimize UC clinical outcomes. Our findings revealed that elevated efferocytosis-associated hub genes (ANXA1, CD93, etc.) expression predicts resistance to UST, likely due to their direct interactions with the drug. This specificity contrasts with their lack of association with responses to TNF-α inhibitors, positioning these genes as predictive biomarkers for UST responsiveness, thereby advancing personalized therapeutic strategies for UC patients receiving UST.

Our findings advance prior work by resolving efferocytosis dynamics at single-cell resolution and integrating cross-species validation. While earlier studies focused on macrophage polarization (22), our data emphasize pathway-specific dysregulation, revealing myeloid-stromal crosstalk as a therapeutic axis. However, limitations exist: the small scRNA-seq cohort may underrepresent rare cell subsets, and DSS-colitis mice inadequately model chronic UC fibrosis. Third, the association between efferocytosis-related hub genes (ANXA1, CD93, etc.) and UST response was derived solely from analysis of the GSE206285 cohort. This single-dataset finding lacks external validation and could not be adjusted for key clinical confounders (e.g., baseline disease severity, prior biologic exposure), necessitating cautious interpretation and prospective validation. Finally, while molecular docking predicted structural complementarity between UST and several hub proteins, these computational results do not establish biological relevance to UST’s primary IL-12/IL-23 blockade mechanism and require experimental validation to determine functional significance. Future studies should validate hub genes in prospective clinical trials controlling for confounders and utilize animal models, such as myeloid-specific Fra-1 knockout in DSS-induced colitis, to assess its impact on M2 polarization, efferocytosis, and disease severity. Additionally, organoid models could elucidate how ANXA1 knockdown affects epithelial repair.

## 5 Conclusion

Efferocytosis, a critical process in resolving inflammation, involves myeloid cells (via “eat me” signals) and stromal cells (via “find me” signals), with M2 macrophages exhibiting superior apoptotic clearance. Efferocytosis dysfunction in UC arises from disrupted myeloid-stromal coordination and M2 macrophage depletion. We identified efferocytosis-associated hub genes (Anxa1, Cd93, Mfge8) that correlate with disease severity but not lesion localization, suggesting universal biomarkers. Notably, these genes predict UST responsiveness but not GLM/IFX/VDZ efficacy, highlighting their potential as UST-specific biomarkers and mechanistic clues for IL-12/IL-23 pathway effects on efferocytosis. These findings underscore efferocytosis modulation as a therapeutic strategy and advocate biomarker-driven approaches to optimize UC treatment outcomes.

## Data Availability

The datasets presented in this study can be found in online repositories. The names of the repository/repositories and accession number(s) can be found below: PXD063633 (PRIDE), https://www.ebi.ac.uk/pride/archive/projects/PXD063633.

## Supplementary material

SUPPLEMENTARY DATA SHEET 1 Technical details of single-cell RNA Sequencing and ASTRAL-DIA Proteomic analysis.

Supplementary Table 1 Complete Ligand-Receptor Interaction by CellChat.

Supplementary Table 2 Comprehensive list of efferocytosis-related genes.

Supplementary Figure 1 Batch effect correction and biological signal preservation in single-cell RNA-seq analysis following Harmony integration. **(A)** T-SNE plot colored by sample origin after Harmony integration. **(B)** T-SNE plot colored by prime cell type. **(C)** T-SNE plot colored by disease status after Harmony integration.

Supplementary Figure 2 Identification of myeloid cell subpopulations.

Supplementary Figure 3 Identification of stromal cell subpopulations.

Supplementary Figure 4 Weighted gene co-expression network analysis (WGCNA) of colitis. **(A)** Determination of soft-threshold power. **(B)** Cluster dendrogram of highly connected genes in key modules.

Supplementary Figure 5 Receiver operating characteristic (ROC) analysis of hub genes for predicting Ustekinumab response. **(A)** Single-gene ROC curve for predicting clinical remission. **(B)** Multi-gene panel ROC for clinical remission. **(C)** Single-gene ROC curve for predicting mucosal healing. **(D)** Multi-gene panel ROC for mucosal healing.

Supplementary Figure 6 Prediction of the binding affinity between ustekinumab and hub genes. **(A)** The Docking model of the UST-ANAX1 protein complex. **(B)** The Docking model of the UST-PANX1 protein complex. **(C)** The Docking model of the UST-ANXA5 protein complex. **(D)** The Docking model of the UST-CD93 protein complex. **(E)** The Docking model of the UST-MFGE8 protein complex. **(F)** The Docking model of the UST-ANAX1 protein complex by SERPINE. All docking models were performed using GRAMM, and the binding affinities were calculated by PDBePISA.
