# Supplementary material for: Multi-omics reveals efferocytosis-related hub genes as biomarkers for ustekinumab response in colitis
Source: Front Immunol. 2025 Sep 19;16:1597528. doi: 10.3389/fimmu.2025.1597528 (PMC12492496; doi:10.3389/fimmu.2025.1597528)
Supplement: SUPPLEMENTARY DATA SHEET 1 — Technical details of single-cell RNA Sequencing and ASTRAL-DIA Proteomic analysis. [file DataSheet1.docx]

**Detailed parameters of Single cell RNA sequencing (ScRNA-seq) data analysis**

**Data processing**

Twelve seurat objects from healthy and UC patients were created using the R package Seurat (version 4.3.0). At this point, doublets were assessed using the scDblFinder (version 1.8.0) and removed. Samples were then pooled together in the same object. Low-quality cells were then filtered out based on mitochondrial RNA percentage and number of genes per cell. Epithelial cells required a less stringent filter of 65% of counts aligned to the mitochondrial genes for quality control. Then, we logarithmically normalized, obtained the highly variable genes, and scaled the counts (default parameters) of each data set using Seurat. Principal component analysis (PCA) was performed. Harmony (version 0.1.1) was used to address the batch effects. We visualized cell clusters in 2D using t-SNE, a standard method to simplify complex gene data, based on key variation patterns (PCs). TSNE also served as a two-dimensional embedding for data visualization. Cluster analysis was performed using the Louvain clustering algorithm.

**Identification of cell types**

The resulting unsupervised 10 clusters were manually categorized into five main cell types: Epithelial cells, T cells, Myeloid cells (mast cells, macrophages, neutrophils, and eosinophils), Stromal cells (endothelial cells, fibroblasts and glia), and B and plasma cells. Immunoglobulin (IG) genes were removed from all the main cell types except B and plasma cells to reduce background noise. Each main cell type was re-processed starting from FindVariableFeatures using the same procedure as for the whole dataset. During the process, doublets were identified through expert annotation of the marker gene lists for each cell cluster and corresponded to clusters with markers from distinct lineages. In brief, The CD3, C1QA, DERL3,or MS4A1-expressing cells from epithelial cells; CD3, C1QA, or EPCAM-expressing cells from the B cells; CD3, C1QA, DERL3, MS4A1 or EPCAM-expressing cells from the stromal cells; and CD3, THY1, DERL3 or MS4A1-expressing cells from the myeloid cells were removed. Then the myeloid cells and stromal cells were re-clustered using an unsupervised Louvain clustering algorithm. The annotation of each subcluster from the main cell type was defined by the marker genes obtained by the FindAllMarkers function and previous study.

**Identification of subpopulation of myeloid cell**

Initial subsetting was based on high expression of canonical markers (C1QA, C1QB, LYZ, MS4A2, TPSAB1).Subtypes were defined by the following marker combinations:

Eosinophils (CLC, IL4, IL13), Mast cells (LTC4S, TPSAB1, TPSB2), Neutrophils (PROK2, CMTM2, CXCL8, AQP9, S100A8), Macrophages (CD68, CD14, C1QA, C1QB). Macrophage subtype refinement were further annotated via combinatorial markers:M0 (pan-macrophage markers+TMSB4X, SELENOP, C1QA, FTL, C1QB), M2(M0 markers + CD163L1, A2M, CD209), IDA(pan-macrophage markers + VCAN, CD300E, FCN1,NRG1, HBEGF), M1(pan-macrophage markers + VCAN, CD300E, FCN1, TNF, IL1B, IL6).

**Identification of subpopulation of stromal cells**

Initial subsetting was based on high expression of canonical markers (ACTA2, ADAMDEC1, CHI3L1, NRXN1, PLVAP, SOX6, VWF).Subtypes were defined by the following marker combinations:

Glial cells (NRXN1, PLP1, S100B, L1CAM, CDH19), Endothelial cells (PLVAP, PECAM1, VWF, GIMAP7, RAMP3), Fibroblast (ADAMDEC1, ABCA8, MEG3, FN1, TNXB). Fibroblast subtype refinement were further annotated via combinatorial markers: Fibroblast2_S4 (IL11, CXCL5, FAP, INHBA, IL24), Fibroblast1_S1/S3 (ADAMDEC1, CP, FABP4, OGN, CCDC80),

**Efferocytosis levels of each cell type**

The four subways of efferocytosis were measured by the AddModuleScore function of the seurat. Heatmap visualization of Efferocytosis levels for different cell types completed by ggplot2.

**Differential Expression Analysis of Subpopulation**

To identify differentially expressed genes (DEGs) in Subpopulation between UC patients and healthy controls, we performed subgroup-specific analysis using the ​FindMarkers function in Seurat (Wilcoxon rank-sum test). DEGs were defined as genes with adjusted p-value (FDR) < 0.05 and absolute log2(fold change) ≥0.25 (corresponding to fold change ≥ 1.19).

**Pseudotime Analysis of macrophages cells**

The Monocle 2 (version2.30.0) were used to order macrophages cells in pseudotime to indicate their developmental trajectories. All distinct macrophages were used to created a CellDataSet object using a negative binomial model. The Monocle 2 run with 2000 most highly variable genes selected from Seurat and default parameters of Monocle after DDRTree dimension reduction and cell ordering. To visualize the ordered cells in the trajectory, we used the plot_cell_trajectory function to plot the minimum spanning tree on the cells.

**Detailed description of Hub genes validation in mice by ASTRAL-DIA Proteomic data**

**Extraction and trypsin treatment of colon proteins in colitis mice**

Proteins from colons were extracted and treated with trypsin. Each colon was lysed in sodium dodecyl titanate (SDT) with NaCl and dithiothreitol (DTT), sonicated, and incubated at 95°C. After centrifugation, the supernatant was alkylated with IAM and precipitated with acetone. The pellet was dissolved and assessed via Bradford assay and SDS-PAGE. For digestion, proteins were diluted in a buffer containing Urea and TEAB, and digested with trypsin at 37°C. The digested proteins were acidified, centrifuged, and loaded onto a C18 desalting column, then lyophilized.

**Vanquish neo UHPLC-astral LC/MS DIA method**

Using a Vanquish Neo UHPLC-Astral LC/MS DIA method, the protein powder was re-dissolved and injected into a UHPLC system with a C18 column. Gradient elution was performed, and the peptides were analyzed by LC-MS/MS on an Orbitrap Astral platform. DIA settings included a full MS scan range of 380-980 m/z and a resolution of 240,000 at 200 m/z.

**Identification and quantitation of hub proteins**

MS/MS data were searched against the UniProt protein database using DIA-NN software, with cysteine alkylation as a fixed modification and methionine oxidation as a variable modification. Results were filtered for a confidence level of 99% or higher, and FDR was controlled below 1%. Retention time correction was applied using iRT standards. Data were analyzed by PCA and CV, with proteins detected in less than two biological replicates removed. Differentially abundant proteins were identified with *P* values < 0.05 and FC > 1.2. The log2 fold change (log2FC) calculation was performed with a pseudocount addition of 1 to avoid extreme values caused by zero expression intensities. The log2FC values were retained to nine decimal places to enhance numerical precision. Subsequently, we identified the differentially expressed proteins of hub genes in mouse colon tissue.
