# Supplementary figures and images for: Multi-omics reveals efferocytosis-related hub genes as biomarkers for ustekinumab response in colitis
Source: Front Immunol. 2025 Sep 19;16:1597528. doi: 10.3389/fimmu.2025.1597528 (PMC12492496; doi:10.3389/fimmu.2025.1597528)

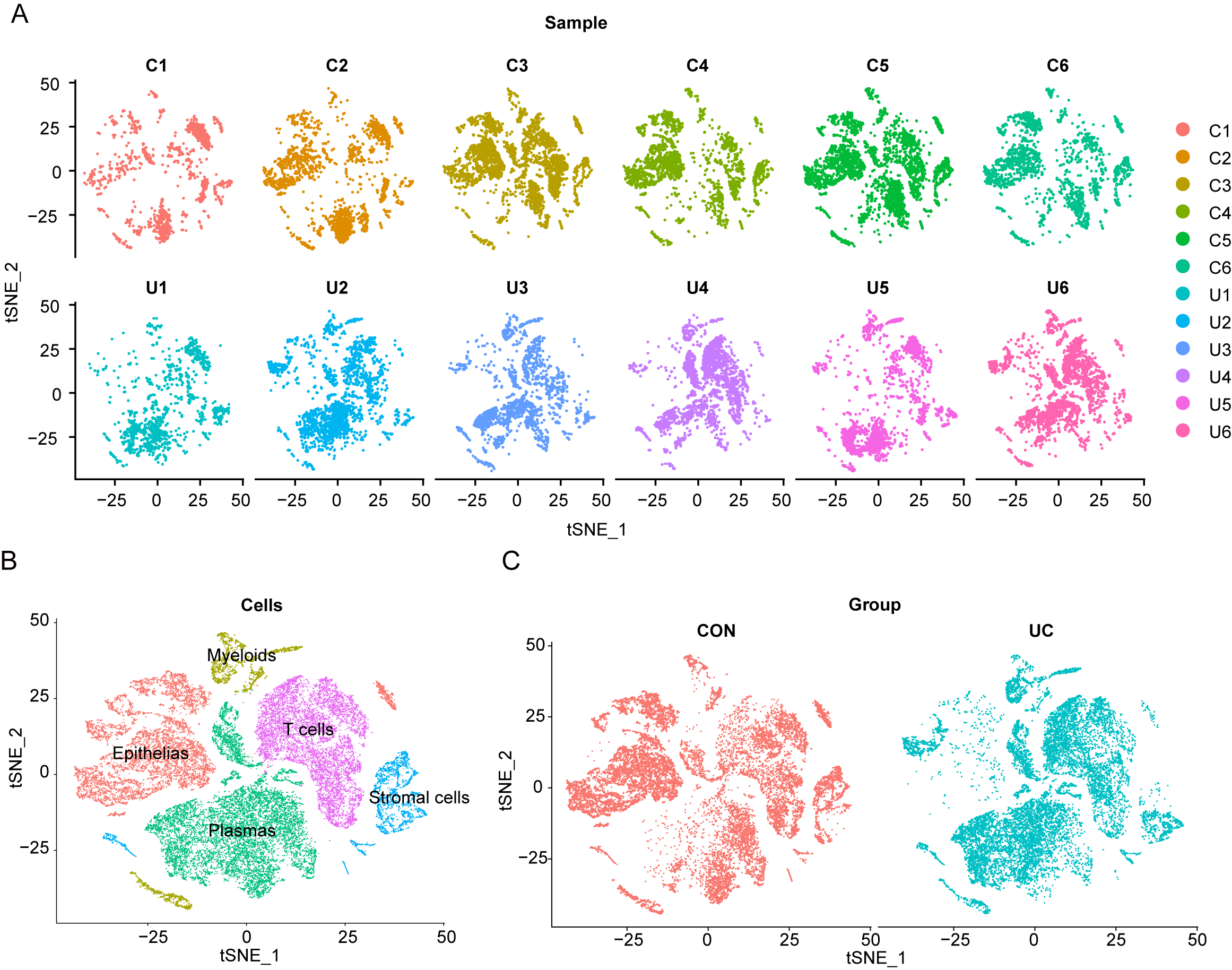

Supplement: Supplementary Figure 1 — Batch effect correction and biological signal preservation in single-cell RNA-seq analysis following Harmony integration. (A) T-SNE plot colored by sample origin after Harmony integration. (B) T-SNE plot colored by prime cell type. (C) T-SNE plot colored by disease status after Harmony integration. [file Image1.tif]

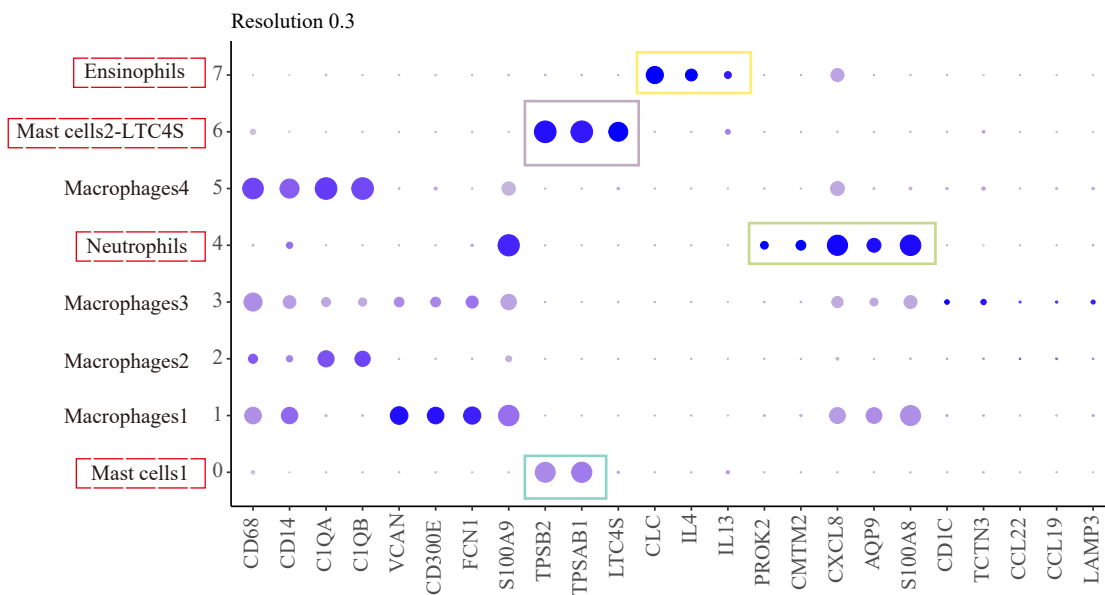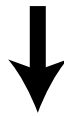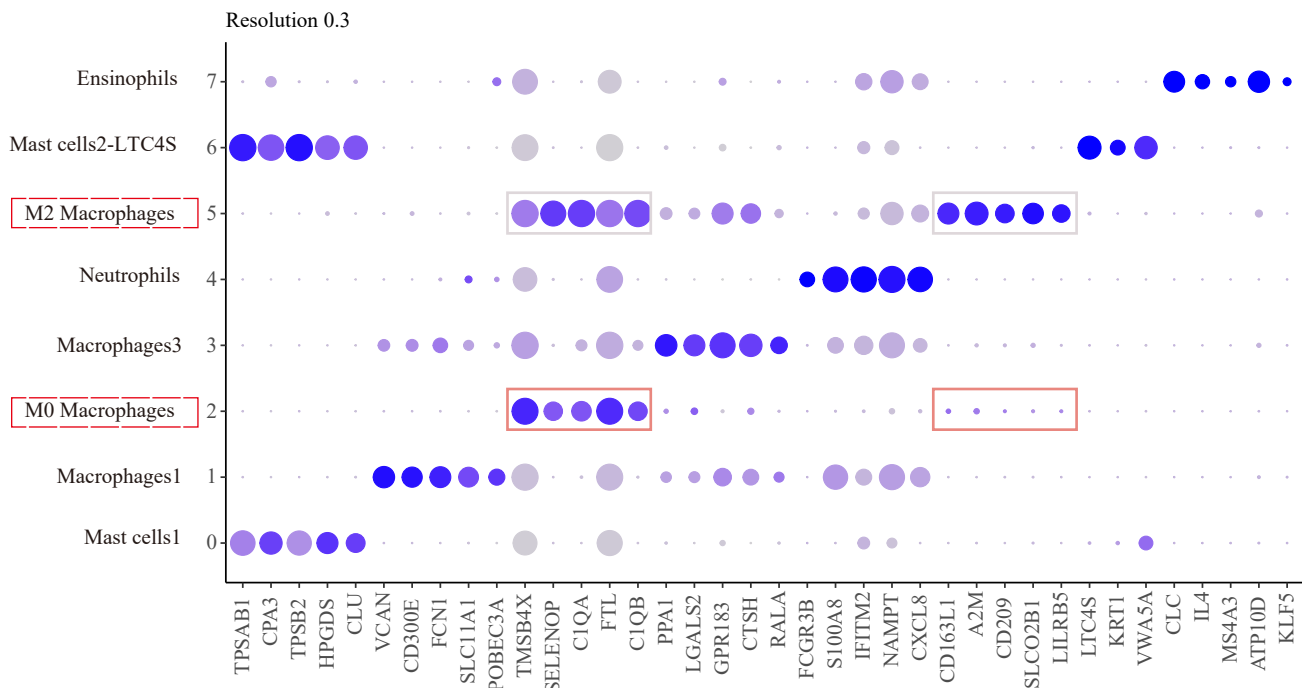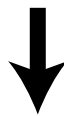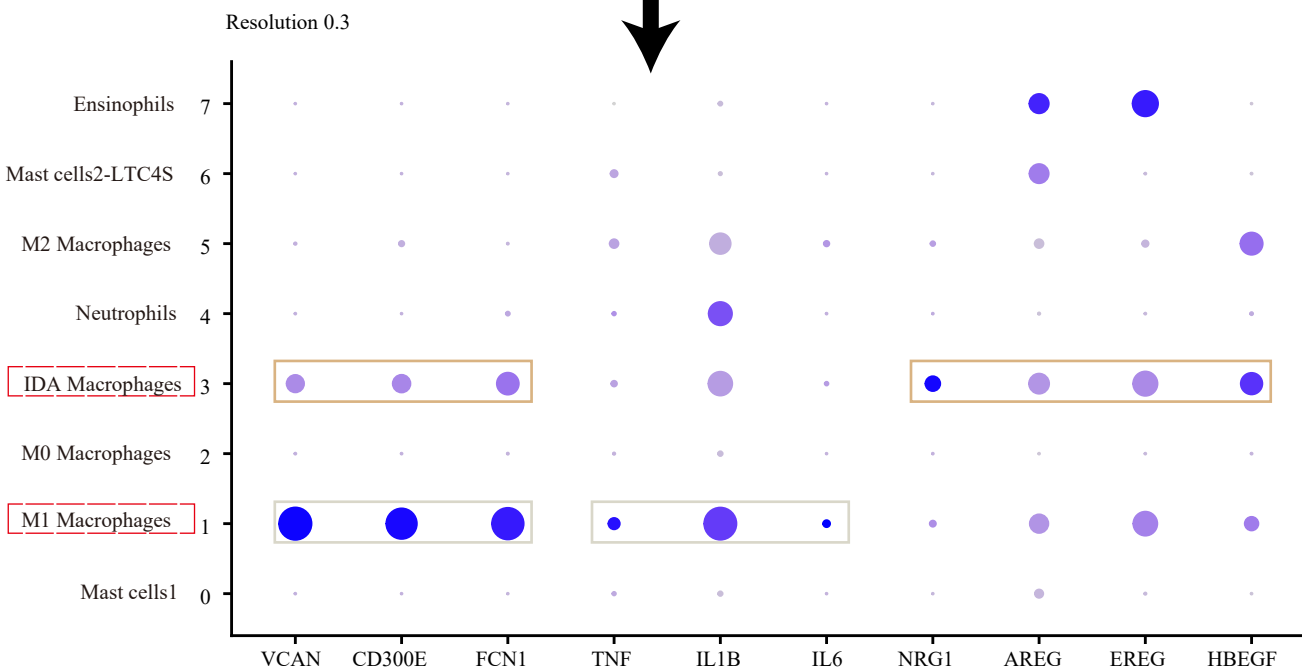

Supplement: Supplementary Figure 2 — Identification of myeloid cell subpopulations. [file Image2.pdf]

Resolution0.1

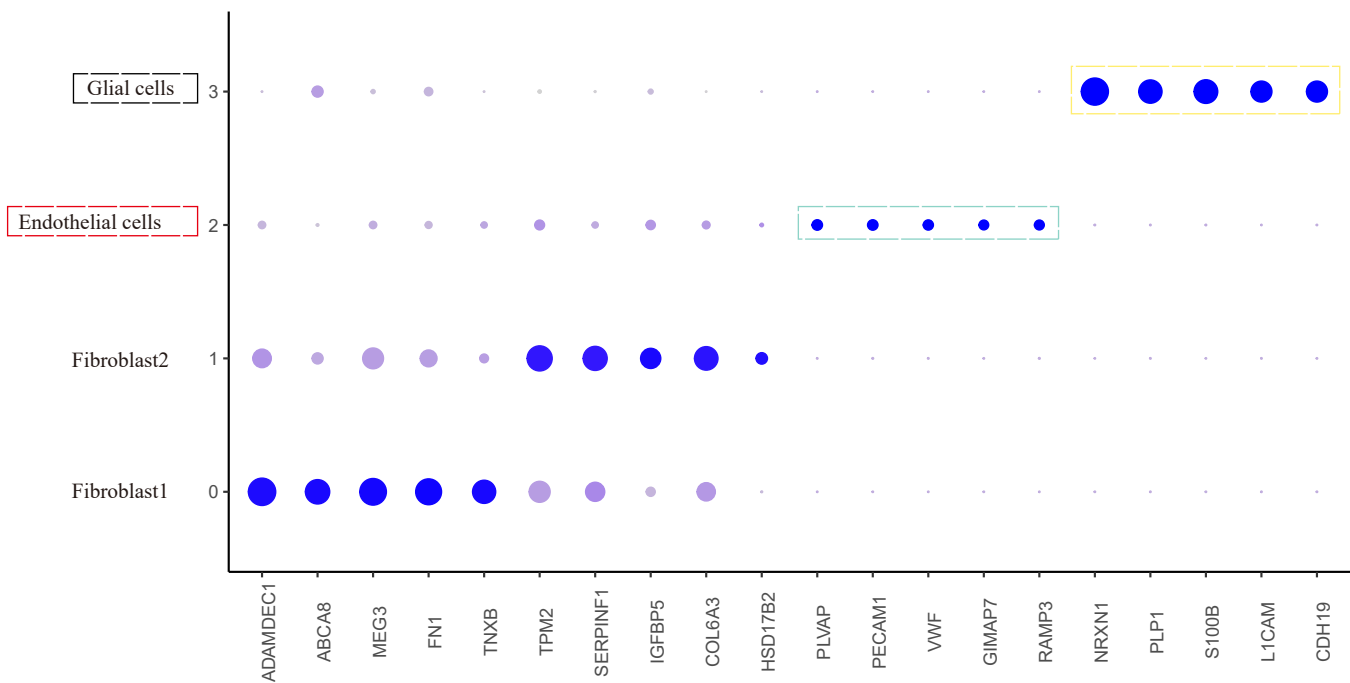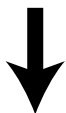

Resolution0.1

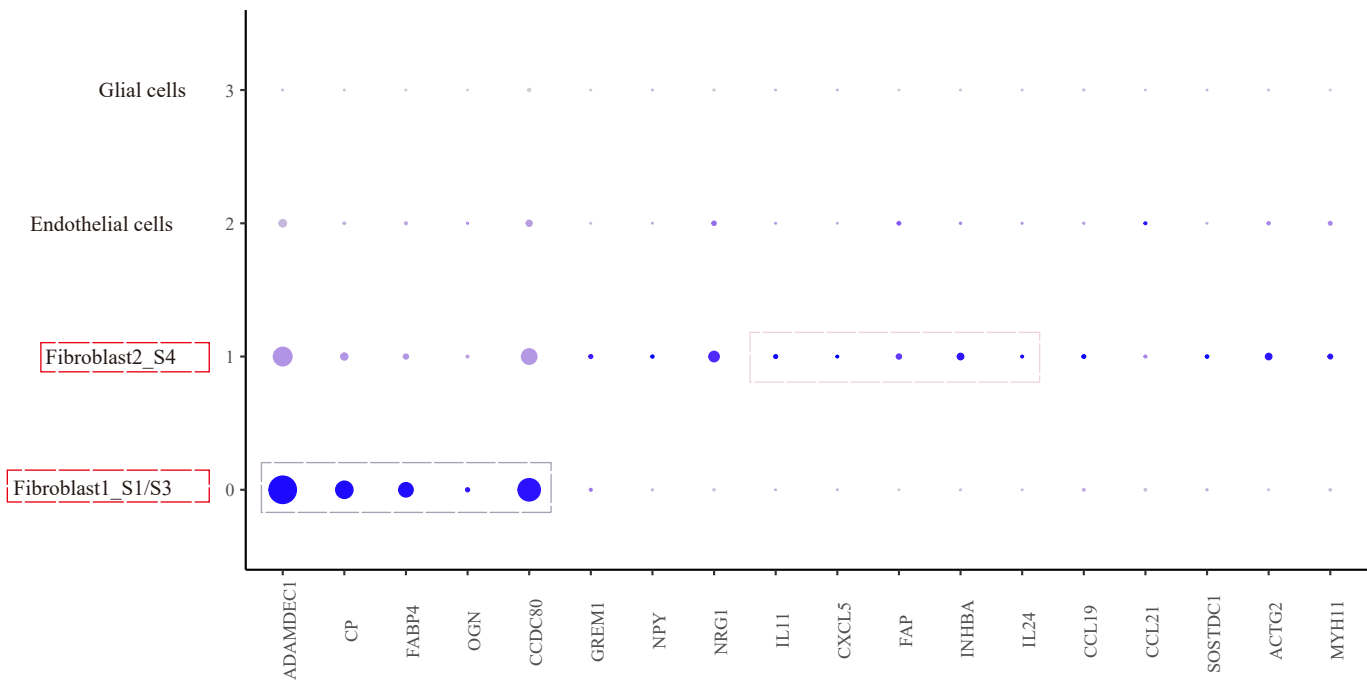

Supplement: Supplementary Figure 3 — Identification of stromal cell subpopulations. [file Image3.pdf]

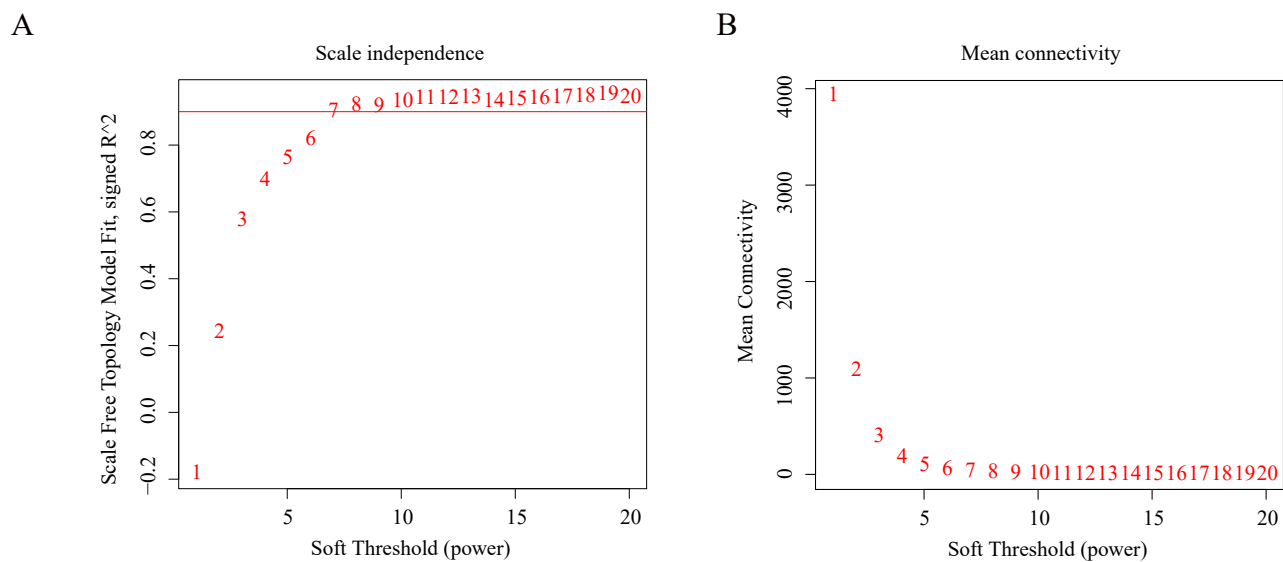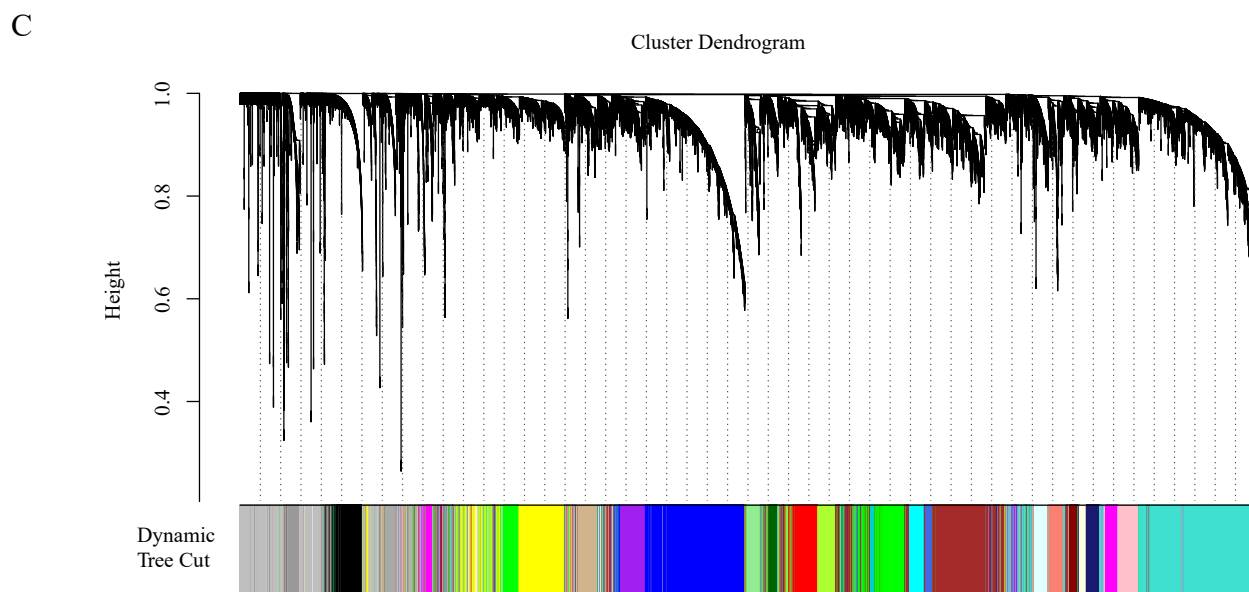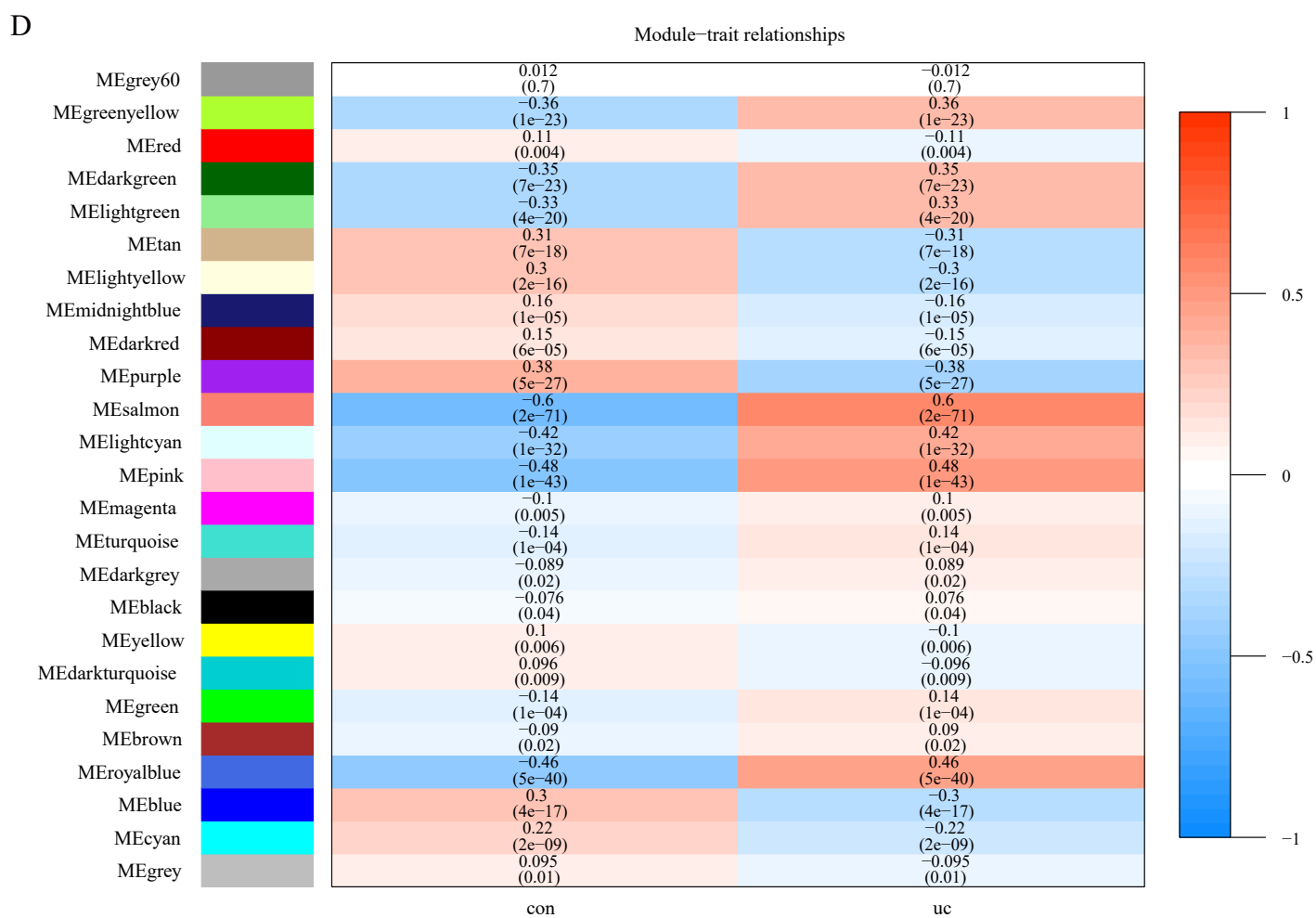

Supplement: Supplementary Figure 4 — Weighted gene co-expression network analysis (WGCNA) of colitis. (A) Determination of soft-threshold power. (B) Cluster dendrogram of highly connected genes in key modules. [file Image4.pdf]

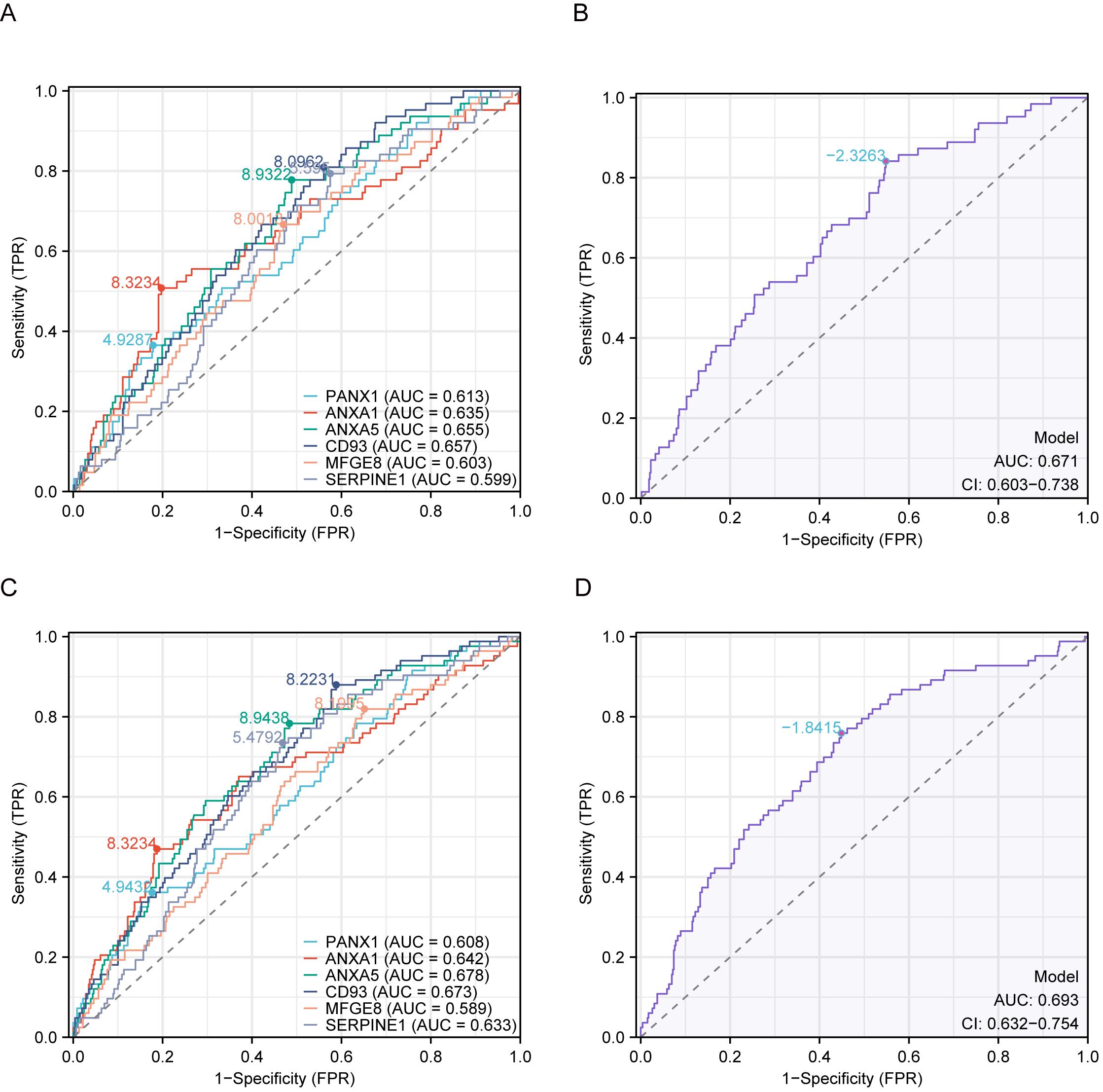

Supplement: Supplementary Figure 5 — Receiver operating characteristic (ROC) analysis of hub genes for predicting Ustekinumab response. (A) Single-gene ROC curve for predicting clinical remission. (B) Multi-gene panel ROC for clinical remission. (C) Single-gene ROC curve for predicting mucosal healing. (D) Multi-gene panel ROC for mucosal healing. [file Image5.tif]

A

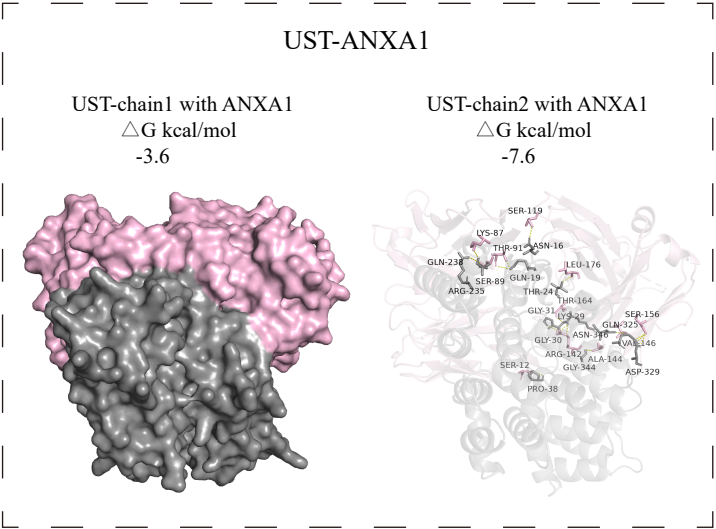

B

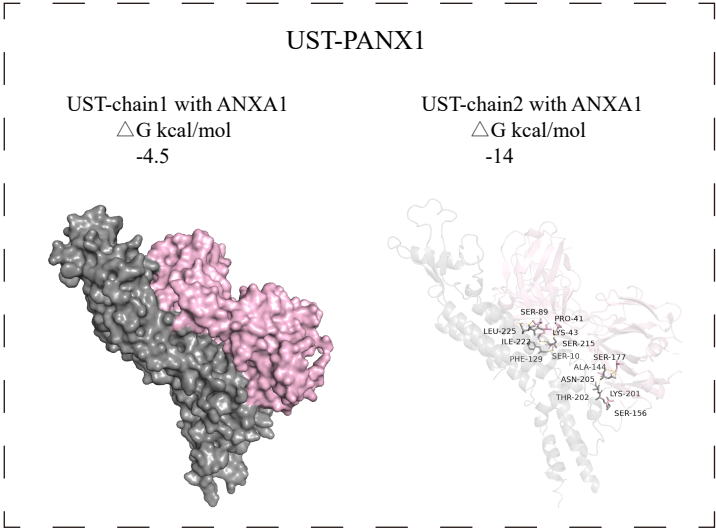

C

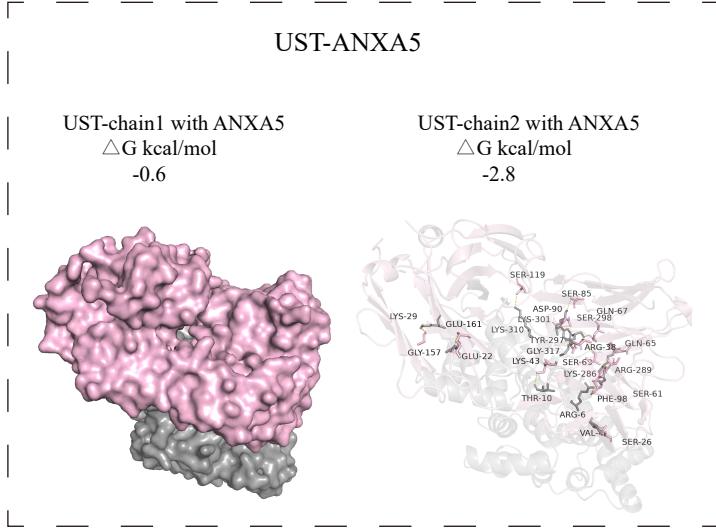

D

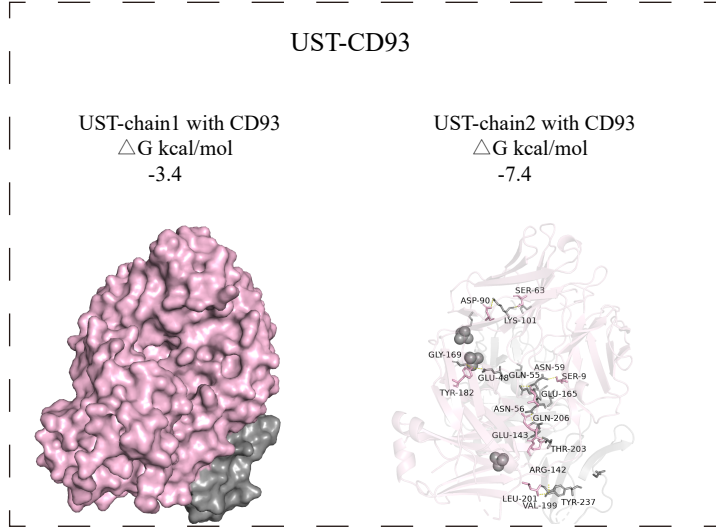

E

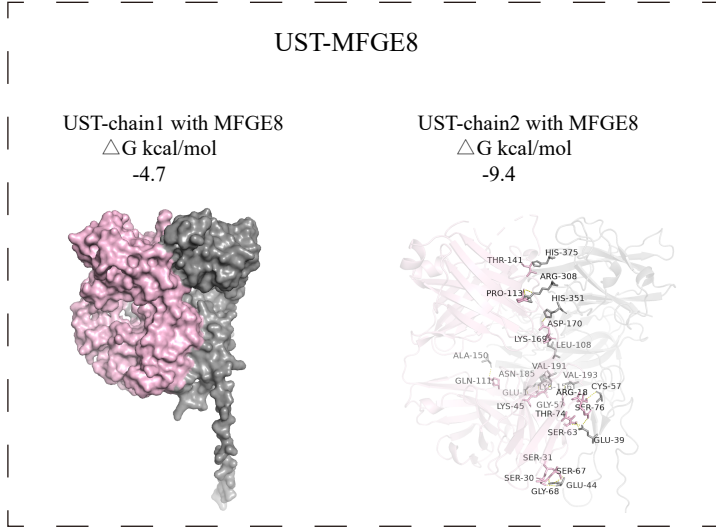

F

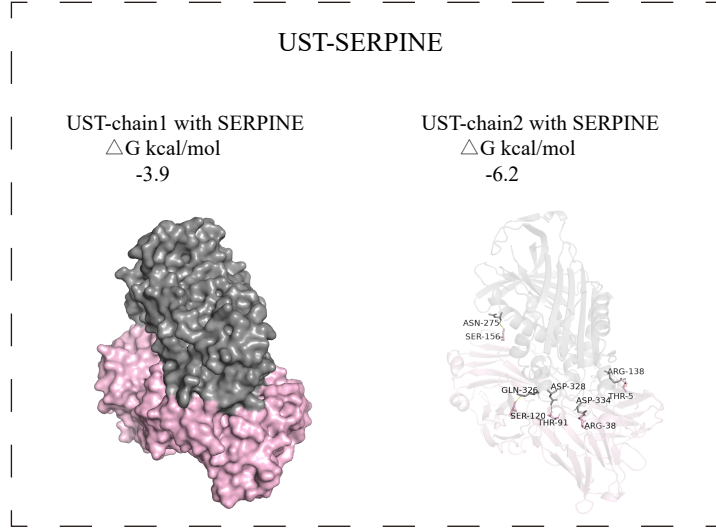

Supplement: Supplementary Figure 6 — Prediction of the binding affinity between ustekinumab and hub genes. (A) The Docking model of the UST-ANAX1 protein complex. (B) The Docking model of the UST-PANX1 protein complex. (C) The Docking model of the UST-ANXA5 protein complex. (D) The Docking model of the UST-CD93 protein complex. (E) The Docking model of the UST-MFGE8 protein complex. (F) The Docking model of the UST-ANAX1 protein complex by SERPINE. All docking models were performed using GRAMM, and the binding affinities were calculated by PDBePISA. [file Image6.pdf]
